# Therapy-induced normal tissue damage promotes breast cancer metastasis

**DOI:** 10.1016/j.isci.2023.108503

**Published:** 2023-11-22

**Authors:** Douglas W. Perkins, Ivana Steiner, Syed Haider, David Robertson, Richard Buus, Lynda O'Leary, Clare M. Isacke

**Affiliations:** 1 The Breast Cancer Now Toby Robins Research Centre, Institute of Cancer Research, 237 Fulham Road, SW3 6JB London, UK

**Keywords:** Cellular therapy, Cell biology, Cancer

## Abstract

Disseminated tumor cells frequently exhibit a period of dormancy, rendering them chemotherapy insensitive; conversely, the systemic delivery of chemotherapies can result in normal tissue damage. Using multiple mouse and human breast cancer models, we demonstrate that prior chemotherapy administration enhances metastatic colonization and outgrowth. *In vitro*, chemotherapy-treated fibroblasts display a pro-tumorigenic senescence-associated secretory phenotype (SASP) and are effectively eliminated by targeting the anti-apoptotic protein BCL-xL. *In vivo*, chemotherapy treatment induces SASP expression in normal tissues; however, the accumulation of senescent cells is limited, and BCL-xL inhibitors are unable to reduce chemotherapy-enhanced metastasis. This likely reflects that chemotherapy-exposed stromal cells do not enter a BCL-xL-dependent phenotype or switch their dependency to other anti-apoptotic BCL-2 family members. This study highlights the role of the metastatic microenvironment in controlling outgrowth of disseminated tumor cells and the need to identify additional approaches to limit the pro-tumorigenic effects of therapy-induced normal tissue damage.

## Highlights

• Prior chemotherapy treatment of mice enhances metastatic colonization and outgrowth
• Chemotherapy treatment induces SASP expression in normal tissues and cultured cells
• Chemotherapy induces fibroblast senescence and sensitivity to navitoclax in culture

## Graphical abstract

**Figure:**
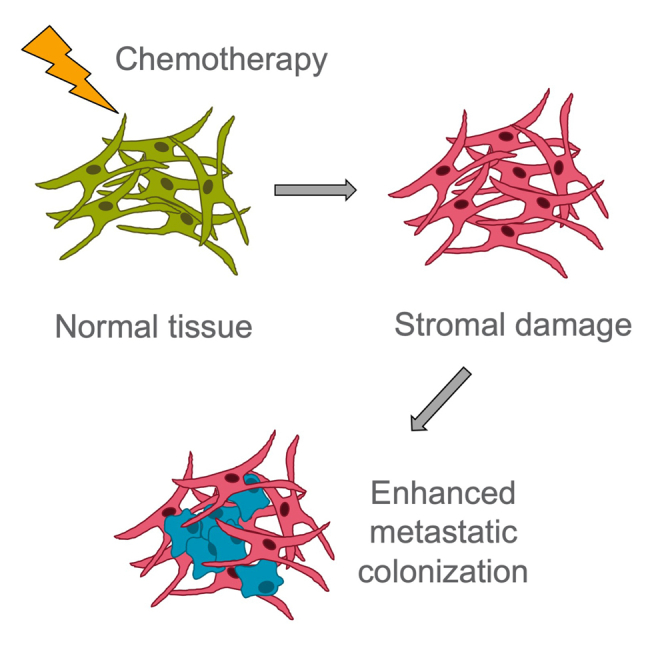

## Introduction

In breast cancer patients, prevention of local recurrence is achieved by surgery and localized radiotherapy, whereas adjuvant systemic chemotherapy or targeted agents are aimed at limiting the outgrowth of disseminated tumor cells (DTCs) at secondary sites. Estrogen receptor-negative (ER^−^) and ER^+^ breast cancers have a different risk of recurrence profiles, with the majority of ER^−^ tumors displaying an early (<5 years after primary diagnosis) relapse and ER^+^ tumors showing a broader profile with a higher incidence of late (>5 years) relapse reflecting the propensity of DTCs to reside in a prolonged period of dormancy prior to re-entering proliferation. Cytotoxic chemotherapy is most effective in targeting proliferating metastatic lesions, whereas dormant DTCs are relatively chemoresistant. In both scenarios, the ratio of tumor cells compared with surrounding secondary site tissue is low; hence the stromal milieu has a profound impact on the fate of disseminated tumor cells and disease outcome. For example, an aging or fibrotic microenvironment has been shown to promote outgrowth of ER^+^ DTCs,^1^^,^^2^ whereas normal tissue radiation exposure enhances metastatic outgrowth in ER^−^ models.^3^ One feature of aging tissues is the accumulation of senescent cells,^4^^,^^5^ which underpin a range of age-related pathological conditions including idiopathic pulmonary fibrosis, atherosclerosis, osteoarthritis, and renal failure.^5^^,^^6^^,^^7^ Further, multiple studies have revealed that increased normal tissue cell senescence can promote tumor progression,^8^^,^^9^ creating a link between aging and cancer.^10^

Here, we have investigated the effect of systemic chemotherapy on the induction of normal tissue damage, including the induction of cellular senescence, and the role of therapy-exposed stromal cells in shaping disease progression.

## Results

### Prior chemotherapy treatment promotes metastatic tumor growth

To test the hypothesis that systemic chemotherapy treatment can prime the host tissue microenvironment, making it a more receptive niche for metastatic tumor development, we employed experimental metastasis assays that discriminate between the effects of the chemotherapy treatment on the stroma from any direct effects on the tumor cells. Naive BALB/c mice were treated with a course of chemotherapy, consisting of three or four doses over two weeks of doxorubicin combined with cyclophosphamide. This schedule reflects the cyclic delivery of chemotherapy in breast cancer patients in the clinic^11^^,^^12^^,^^13^^,^^14^ and anti-tumor efficacy in syngeneic models (Figure S1). Mice were then given a 7–10 days recovery period chosen to allow for clearance of the chemotherapeutic agents^15^^,^^16^ and chemotherapy-induced apoptotic cells^17^ and for the repopulation of circulating lymphocytes.^18^

**Figure 1:**
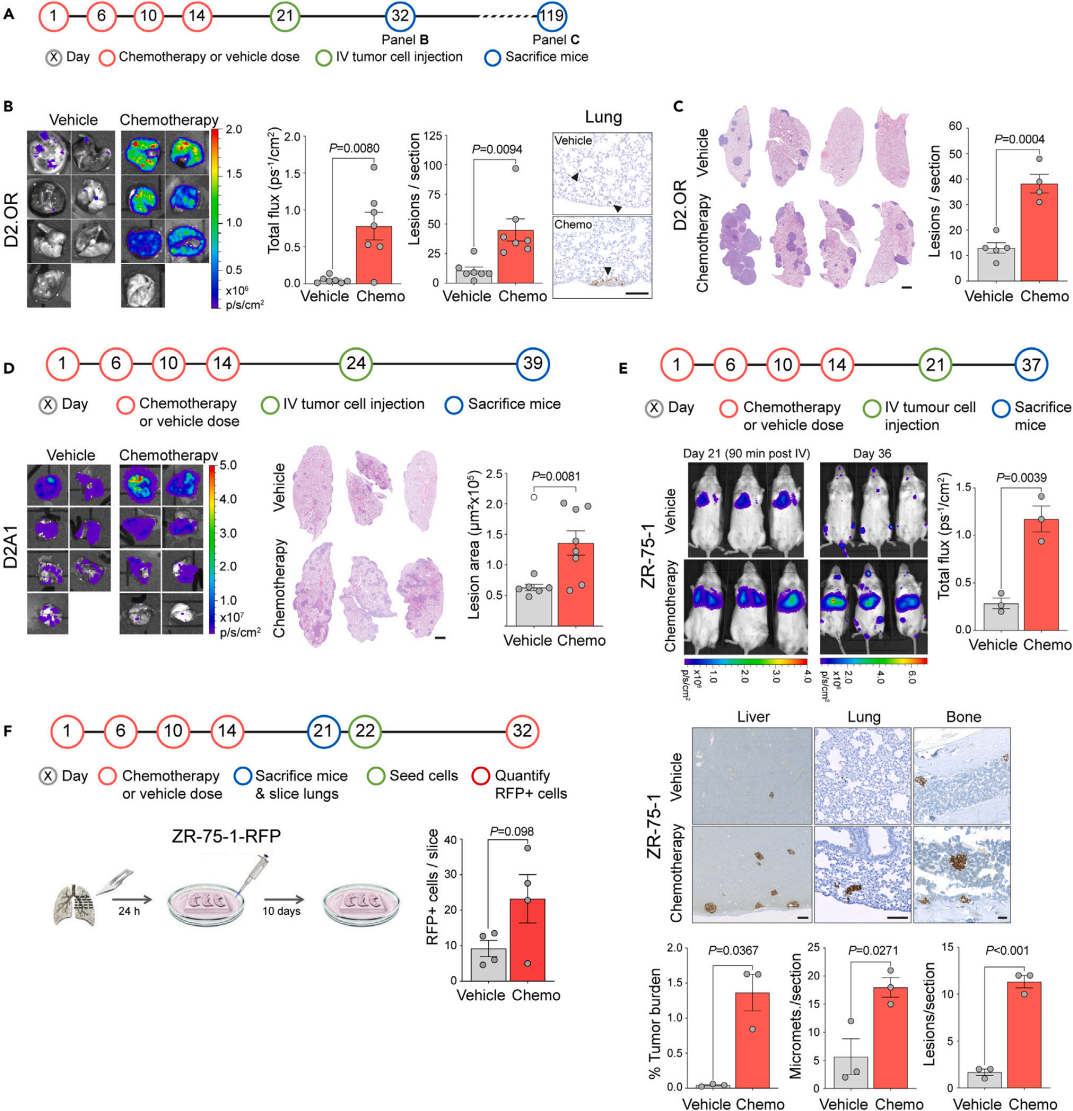
Prior chemotherapy treatment increases metastatic colonization (A) Schedule of chemotherapy (doxorubicin 2.7 mg kg^−1^ and cyclophosphamide 43 mg kg^−1^) or vehicle treatment and subsequent intravenous (IV) tumor cell inoculation. (B) BALB/c mice (n = 7 per group) were treated with 4 doses of combination chemotherapy or vehicle as indicated. On day 21, mice were inoculated via the tail vein with 1 × 10^6^ D2.OR-Luc tumor cells. Mice were sacrificed 11 days after inoculation (day 32). Shown are *ex vivo* IVIS images of the lungs, quantification of IVIS signal in the lungs (mean ± SEM, Welch’s t test), quantification of the mean number of metastatic lesions from three lung sections per mouse (mean ± SEM, Welch’s t test), and representative immunohistochemical images of luciferase staining (scale bar, 125 μm). Black arrowheads indicate single disseminated tumor cells (vehicle) or macrometastatic deposits (chemotherapy). (C) BALB/c mice (n = 4 or 5 per group) were treated with a schedule of chemotherapy or vehicle as outlined in (A). On day 21, mice were inoculated via the tail vein with 1 × 10^6^ D2.OR-Luc tumor cells. Mice were sacrificed 98 days after inoculation (day 119). Shown are representative H&E-stained sections of the lungs (scale bar, 1 mm) and quantification of the mean number of metastatic lesions from three lung sections per mouse (mean ± SEM, Student’s t test). (D) BALB/c mice (n = 7 or 8 per group) were treated as indicated. On day 24, mice were inoculated via the tail vein with 4 × 10^5^ D2A1-Luc tumor cells. Mice were sacrificed 15 days after inoculation (day 39). Shown are *ex vivo* IVIS images of the lungs, representative H&E-stained lung sections (scale bar, 1 mm), and quantification of the mean size of the lung metastatic lesions in two lung sections per mouse (mean values per mouse ±SEM, Welch’s t test). One mouse in the vehicle group was determined to be a statistical outlier, indicated by a hollow data point. (E) NOD Rag gamma (NRG) mice (n = 3 per group) were treated with a schedule of chemotherapy or vehicle as illustrated in the timeline. Mice were implanted subcutaneously with a 0.36 mg estrogen pellet on day 17. On day 21, 2 × 10^6^ ZR-75-1-Luc human breast tumor cells were inoculated via the tail vein and IVIS imaged after 90 min. Fifteen days after tumor cell inoculation (day 36), mice were IVIS imaged *in vivo* (mean ± SEM, Student’s t test), sacrificed 24 h later (day 37), and percentage of metastatic burden based on lamin A/C staining was quantified in three liver sections (mean ± SEM, Welch’s t test; scale bar, 250 μm), three lung sections, and two bone sections (mean ± SEM, Student’s t tests; scale bar, 100 μm) per mouse. (F) BALB/c mice (n = 2 per group) were treated with chemotherapy or vehicle as indicated. On day 21, mice were sacrificed and agarose-perfused lungs sliced and cultured on an inert matrix in M199 medium for 24 h before 1,000 ZR-75-1-RFP human breast cancer cells were seeded onto the slices and incubated for a further 10 days. Two lung slices per mouse were imaged by confocal microscopy and the number of RFP^+^ tumor cells per slice quantified (±SEM, Student’s t test).

After the recovery period, mice were inoculated intravenously with tumor cells. We chose mouse mammary tumor or human breast cancer lines which show limited metastatic outgrowth to model the scenario of non-proliferative disseminated cells in the secondary sites.^19^^,^^20^^,^^21^ Initial experiments were performed with the D2.OR cell line which exhibits a dormant-like phenotype *in vivo*.^19^^,^^22^^,^^23^ Eleven days after tumor cell intravenous inoculation into syngeneic BALB/c mice, single tumor cells were found scattered throughout the lung tissue of vehicle-treated mice, whereas discrete tumor lesions were readily detectable in the mice receiving prior chemotherapy (Figures 1A and 1B). By 98 days after tumor cell inoculation, this translated into a significantly increased lung tumor burden in chemotherapy-treated mice (Figure 1C). To validate these findings, this experiment was repeated using the related D2A1 cell line.^22^^,^^23^ Metastatic lesions were detectable in the vehicle-alone group 15 days after intravenous inoculation; however, the size of the lesions significantly increased in the mice receiving prior chemotherapy treatment, indicating more efficient outgrowth (Figure 1D).

Finally, the experimental approach was repeated using immunocompromised NOD RAG gamma (NRG) mice inoculated with human ER^+^ ZR-75-1 breast cancer cells, a cell line exhibiting a dormant phenotype in *in vivo* models.^2^^,^^24^ Again there was a striking enhancement of metastatic colonization in the mice that had received prior chemotherapy. While intravenously injected cells initially disseminated to the lungs, the majority of subsequent metastatic burden manifested in the liver (Figure 1E), with lung and bone metastasis also significantly increased. These effects were recapitulated in *ex vivo* assays with a significant increase in the number of viable ZR-75-1 cells surviving 10 days after seeding onto *ex vivo* organotypic lung slices derived from chemotherapy-treated, compared with vehicle-treated, mice (Figure 1F).

### Chemotherapy treatment causes normal tissue damage

**Figure 2:**
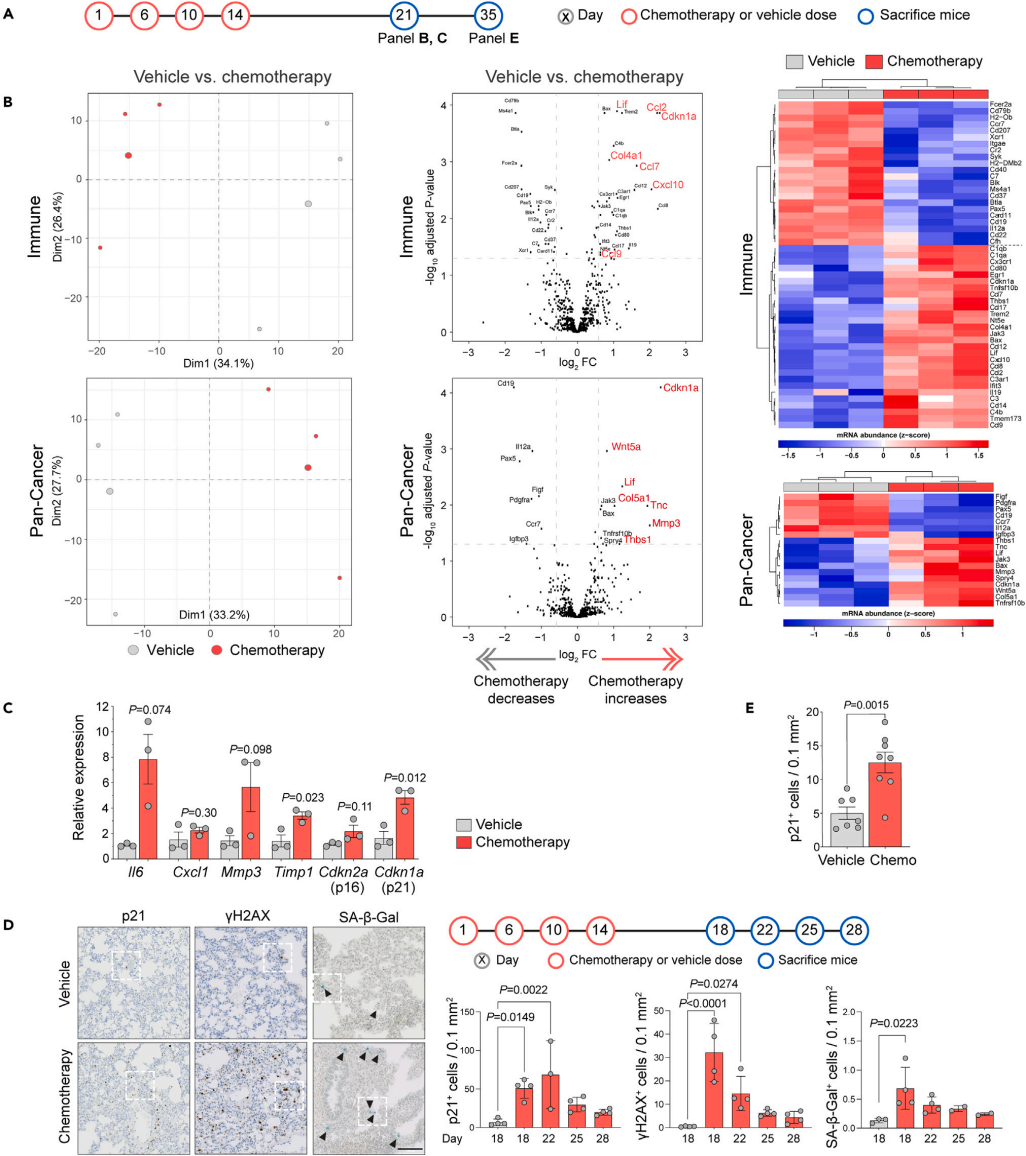
Characterization of the chemotherapy-treated mouse lung (A) Schedule of the chemotherapy or vehicle treatment and subsequent tissue collection time points. (B) BALB/c mice (n = 3 per group) were treated with four doses of chemotherapy or vehicle as illustrated in (A). On day 21, mice were sacrificed and the lungs snap-frozen prior to RNA extraction. Gene expression profiling using the NanoString mouse PanCancer Immune (upper) or PanCancer Pathways (lower) panels. Left panels, principal-component analysis; middle panels, volcano plot showing differentially expressed (DE) genes in chemotherapy- versus vehicle-treated groups. Genes with absolute log_2_ fold change ≥0.585 and FDR adjusted p value <0.05 were considered significant and shown in the heatmap (right panels). Genes highlighted in red are discussed in the text. (C) Expression of the selected DE genes indicated in (B) measured by RT-qPCR (mean ± SEM, Student’s t test except *Il6* Welch’s t test). (D) BALB/c mice (n = 4 per group) were treated with four doses of chemotherapy or vehicle as illustrated in the timeline and sacrificed on days 18, 22, 25, or 28 (n = 4 per group). Lungs were removed, and right lobes were fixed in formalin for immunohistochemistry, whereas left lobe was embedded in OCT and snap-frozen for SA-β-Gal staining. (Left panel) Representative images of p21 (Abcam) and γH2AX staining and SA-β-Gal activity (day 18, vehicle; day 22, chemo; scale bar, 100 μm). Arrowheads indicate blue SA-β-Gal^+^ cells. (Right panels) Quantification of staining (mean ± SD, one-way ANOVA). Higher power images of indicated areas are shown in Figure S2C. (E) BALB/c mice (n = 7 or 8 per group) were treated as illustrated in (A) and sacrificed on day 35. p21 staining (Dako) in lung tissue was quantified (mean values ± SEM, Student’s t test).

Using freshly extracted RNA from lungs of mice sacrificed 7 days after the last chemotherapy treatment (day 21) (Figure 2A), gene expression profiling was performed using the NanoString mouse PanCancer Immune and PanCancer Pathways panels (Figure 2B; Tables S1 and S2). By principal-component analysis (PCA), the vehicle-treated mice clustered separately from the chemotherapy-treated mice, implying global differences across the 750 target gene panels. The significantly upregulated genes in the chemotherapy-treated samples clustered in pathways associated with pro-inflammation, apoptosis, cytokines and chemokines, and pro-cancer progression, indicating that chemotherapy treatment creates a favorable, tumor-promoting microenvironment. These include the interleukin-6 (IL-6) family cytokine *Lif*, which promotes cell proliferation via STAT3 signaling and has been reported to promote breast cancer metastasis;^25^^,^^26^ *Cxcl10*, reported to enhance breast cancer lung metastasis^27^^,^^28^; the pro-tumorigenic factor *Wnt5a*;^29^^,^^30^ *Ccl2* (MCP-1), a potent macrophage chemoattractant, reported to play a role in cancer progression^15^^,^^31^^,^^32^; and the related factors *Ccl7* (MCP-3), *Ccl8* (MCP-2), *Ccl12* (MCP-5), *Cd14*, and *Cx3cr1*.^33^^,^^34^^,^^35^^,^^36^ In contrast, downregulated genes clustered primarily in humoral immunity pathways such as the B cell surface antigens *Cd79b*, *Ms4a1*, and *Cd19* and the B cell lineage-specific *Pax5*. Analysis of the immune cell abundance using the NanoString Immune data confirmed a significantly reduced abundance in B cells and an increased abundance of dendritic cells in chemotherapy-treated lungs (Figure S2A). This analysis also identified a small but significant decrease in T cell abundance but no differences in any T cell subsets. Similarly, immunohistochemical staining of lungs taken between 1 and 27 days after the last dose of chemotherapy revealed no notable changes in the number of CD8- or CD4-positive cells (Figure S2B).

It has been reported that normal tissue senescence is elevated following systemic chemotherapy treatment.^37^ Despite being long-lived and non-proliferative, senescent cells are far from static and inert. Rather, they are highly metabolically active and have elevated expression of the senescence-associated secretory phenotype (SASP), a diverse cocktail of factors responsible for many of their non-cell-autonomous functions.^38^^,^^39^^,^^40^ In both NanoString panels, a top hit within the significantly upregulated genes in chemotherapy-treated mice was *Cdkn1a* (p21), a marker of DNA-damage-induced cell-cycle arrest and cellular senescence,^41^^,^^42^^,^^43^ along with elevated expression of SASP genes such as *Mmp3*, *Cdkn2a*, *Il6*, and *Cxcl1* and *Cxcl10*, and upregulation of *Ifit3* gene implicated in inducing p21 expression.^44^ RT-qPCR analysis confirmed a significantly increased expression of canonical SASP factors in the lung tissue of the chemotherapy-treated mice (Figure 2C), whereas staining of lung sections revealed elevated numbers of p21^+^ cells in chemotherapy-treated lungs taken from mice sacrificed between day 18 and 28 (Figures 2D and S2C). Similarly, chemotherapy-induced activation of the DNA damage response (DDR), manifested by the phosphorylation of DNA damage mediator proteins such as γH2AX, was evident in the mouse lungs after 18 and 22 days (Figure 2D). In an independent experiment where mice were sacrificed 21 days following chemotherapy treatment (day 35), an elevated number of p21^+^ cells were detected persisting in the lungs (Figure 2E). Finally, assessing fresh frozen sections for senescence-associated β-galactosidase (SA-β-Gal) activity as alternative marker of cellular senescence revealed an elevated number of SA-β-Gal^+^ cells in chemotherapy-treated lungs (Figures 2D and S2C). However, it is notable that a much smaller number of SA-β-Gal^+^ cells were detected compared with p21^+^ cells, indicating either that SA-β-Gal^+^ cells are cleared within the 7 days after chemotherapy treatment and/or that the tolerable chemotherapy regime used *in vivo* is insufficient to drive stromal cells from an SASP-positive state into senescence.

### Chemotherapy-treated fibroblasts promote tumor cell growth

Among the increased levels of SASP and pro-tumorigenic factors in the gene expression analysis were extracellular matrix remodeling and fibrosis genes (Figure 2B), including collagens type Va1 (*Col5a1*) and type IVa1 (*Col4a1*), *Mmp3, Thbs1* (thrombospondin1), and *Tnc* (tenascin C), profiles associated with activated fibroblasts.^45^ Tissue fibrosis is associated with increased tumorigenicity in multiple cancer types.^2^^,^^46^^,^^47^

**Figure 3:**
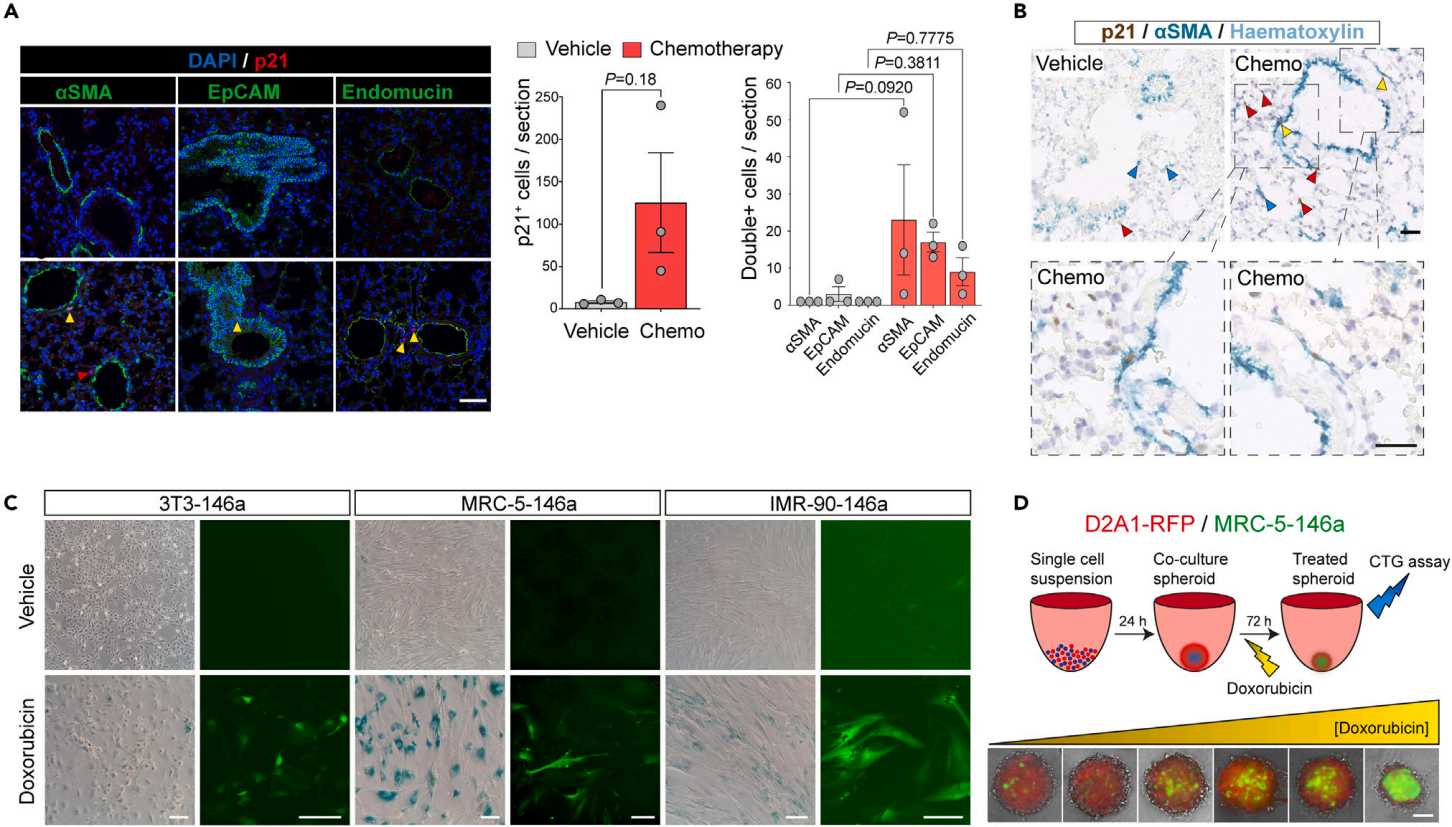
Fibroblast responses to chemotherapy treatment (A) BALB/c mice (n = 3 per group) were treated with three doses of chemotherapy or vehicle on day 1, 5, and 9 and sacrificed on day 16. FFPE lung sections were stained for p21 (red; Dako) and DAPI (blue) and either α-smooth muscle actin (α-SMA), EpCAM, or endomucin (green); scale bar, 50 μm. Red arrowheads indicate p21 single-positive cell; yellow arrowheads indicate p21+ αSMA+ cells. Staining was quantified by counting all p21^+^ cells per section (middle panel, mean ± SEM, Welch’s t test), then counting cells positive for p21 and either α-SMA, EpCAM, or endomucin (right panel, mean ± SEM, one-way ANOVA). (B) Lung FFPE sections from BALB/c mice sacrificed 7 days following the final chemotherapy treatment or 1 day following the final vehicle treatment were stained for p21 (Dako, brown) and αSMA (cyan) and counterstained with hematoxylin. Blue arrowheads indicate p21^−^ αSMA^+^ single-positive cells; red arrowheads indicate p21^+^ αSMA^−^ cells; yellow arrowheads indicate p21^+^ αSMA^+^ positive cells. Left panels; scale bar, 50 μm. Lower panels; higher magnification views of indicated regions; scale bar, 25 μm. (C) Human lung MRC-5-146a fibroblasts, human lung IMR-90-146a fibroblasts, and mouse 3T3-146a fibroblasts were treated with 170 nM doxorubicin for 24 h, then drugs were removed; 20 (3T3-146a) or 21 (MRC-5-146a and IMR-90-146a) days later parallel wells were fixed and stained for SA-β-Gal or expression of the GFP senescence reporter was visualized under a fluorescence microscope (scale bar, 200 μm). Vehicle-treated fibroblasts were seeded 3 days prior to staining or imaging alongside chemotherapy-treated fibroblasts. (D) 1,000 D2A1-RFP mouse tumor cells were co-seeded with 1,000 MRC-5-146a human fibroblasts into ultra-low attachment U-bottom 96-well plates to form co-culture spheroids. After 24 h, spheroids were treated with doxorubicin (0–850 nM); 72 h later, spheroids were imaged (scale bar, 100 μm).

To elucidate whether damaged/activated fibroblasts are present in non-tumor-bearing chemotherapy-treated mice, lung sections from vehicle or chemotherapy-treated mice were co-stained for p21 alongside cell-type markers for myofibroblasts (alpha-smooth muscle actin, αSMA), epithelial cells (EpCAM), and endothelial cells (endomucin) (Figure 3A). Consistent with the data presented in Figures 2C–2E showing significantly increased p21^+^ cells and *Cdkn1a* (p21) expression, a trend for increased p21^+^ cells was observed in lung tissue of the chemotherapy-treated mice (Figure 3A, middle panel). Because p21^+^ cells in the lungs of vehicle-treated mice are scarce, very few double-positive (i.e., stained for both p21 and either αSMA, EpCAM, or endomucin) cells of any type were identified. In contrast, in the chemotherapy-treated lung tissue, p21^+^ cells were readily identified, with the greatest increase being in p21^+^ αSMA^+^-stained cells (Figure 3A, right panel). The identification of p21^+^ αSMA^+^ cells in chemotherapy-treated lung tissue was validated in immunohistochemical staining of lung sections from an independent experiment. The majority of αSMA staining in vehicle-treated lungs were localized to the vasculature, consistent with αSMA expression by pericytes and perivascular-localized fibroblasts. By contrast, in chemotherapy-treated lungs, p21^+^ αSMA^+^ positive cells were detected, both associated with, and distant from, the vasculature (Figures 3A and 3B).

To investigate the response of fibroblasts to chemotherapy treatment *in vitro*, primary human or established mouse fibroblasts were treated for 24 h with doxorubicin, followed by culture in the absence of doxorubicin, resulting in a robust cell-cycle arrest and accumulation of SA-β-Gal^+^ cells (Figure 3C). In an independent approach, fibroblasts were transfected with a senescence reporter construct pmiR146a-GFP,^48^ in which GFP expression is driven by the senescence-associated microRNA146a promoter. Doxorubicin treatment resulted in increased expression of the senescence reporter, which is maintained for up to 20 days following drug withdrawal (Figure 3C). To model this in more complex *in vitro* models, RFP-tagged D2A1 mouse mammary tumor cells were admixed with human miR146a-transfected MRC-5 fibroblasts and co-seeded into ultra-low attachment U-bottom 96-well plates to form 3D co-culture spheroids (Figure 3D). Treatment with increasing concentrations of doxorubicin resulted in effective elimination of the tumor cells, whereas the admixed fibroblasts were induced to express markers of senescence and persist until the end of the assay.

**Figure 4:**
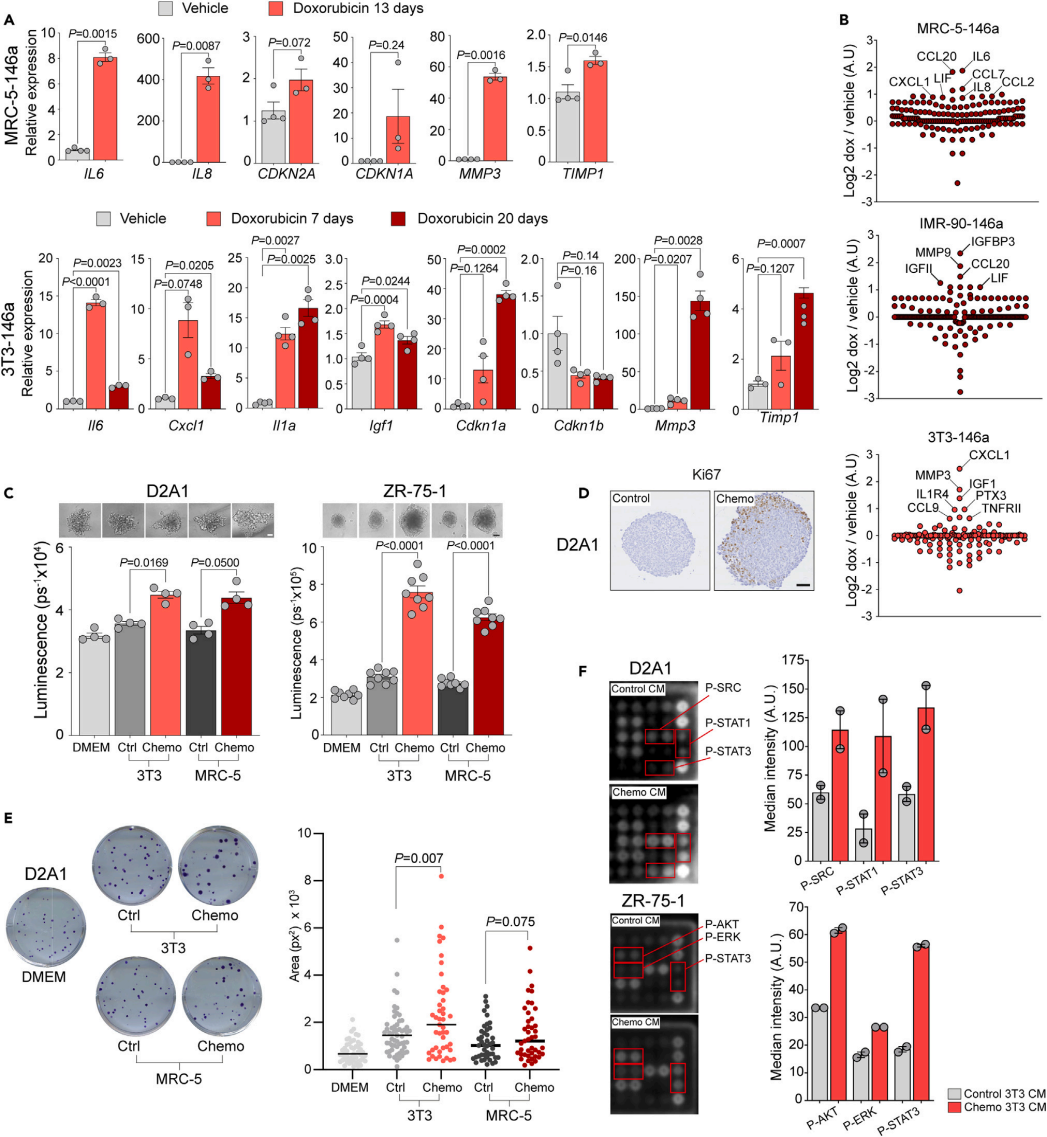
*In vitro* characterization of chemotherapy-treated fibroblasts (A) Human MRC-5-146a or mouse 3T3-146a fibroblasts were treated with 170 nM doxorubicin or vehicle for 24 h. At the indicated time point after drug withdrawal RNA was extracted for RT-qPCR analysis (mean ± SEM, Student’s t test [MRC-5-146a] or one-way ANOVA [3T3-146a] for individual genes). (B) Human MRC-5-146a, human IMR-90-146a, or mouse 3T3-146a fibroblasts were treated with doxorubicin or vehicle as described in (A). Serum-free CM collected 20 days after doxorubicin withdrawal or from vehicle-treated fibroblasts was analyzed using G2000 cytokine arrays. (C) 2,000 ZR-75-1 or D2A1 tumor cells per well were seeded into ultra-low attachment U-bottom 96-well plates in DMEM plus 2% FBS. After 24 h, spheroids were incubated with DMEM supplemented with 2% FBS or CM from control or chemotherapy-treated 3T3-146a mouse fibroblasts or MRC-5-146a human fibroblasts supplemented with 2% FBS. After 4 days (ZR-75-1; n = 4 spheroids per condition) or 6 days (D2A1; n = 8 spheroids per condition), spheroid viability was measured using the CellTiter-Glo assay (mean ± SEM, one-way ANOVA; one-way ANOVA with Dunnett’s correction for multiple comparisons—ZR-75-1). Equivalent results were obtained in 2 (ZR-75-1) or >3 (D2A1) independent experiments. Representative phase contrast images of spheroids are shown above (scale bar, 100 μm). (D) D2A1 spheroids were treated with control or chemotherapy-treated 3T3-146a CM as described in (C) and collected after 4 days of incubation. FFPE sections from embedded spheroids were stained for Ki67 (scale bar, 50 μm). (E) 100 D2A1 tumor cells per well were seeded into 6-well plates in DMEM plus 2% FBS or in CM from control or chemotherapy-treated 3T3-146a mouse fibroblasts or MRC-5-146a human fibroblasts supplemented with 2% FBS. 14 days later, plates were stained with crystal violet. (F) Colony size was quantified using QuPath,^49^ unpaired t tests. (F) 12,500 ZR-75-1 or D2A1 tumors cells per well were seeded into U-bottom 96-well plates in DMEM plus 2% FBS and incubated for 48 h. Spheroids were serum-starved for 1 h and then incubated for 30 min at 37°C with serum-free control or chemotherapy-treated 3T3-146a fibroblast CM for 30 min. Protein was extracted and analyzed on phosphoprotein antibody arrays slides (n = 2 slides per condition, n = 2 phosphoprotein antibody spots per array); ZR-75-1, 112.5 μg total protein per sample; D2A1, 30 μg total protein per sample. (Left panels) Cropped images of the relevant portion of the array. (Right panels) Bar graphs show mean quantification of the two replicate target antibody spots in the two replicate slides (mean ± SEM).

Chemotherapy-treated primary human lung fibroblasts and mouse fibroblast lines show a robust upregulated expression of canonical SASP transcripts (Figure 4A), accompanied by increased secretion of SASP factors into the culture medium (Figure 4B). Incubation of mouse or human tumor spheroids with conditioned medium harvested from doxorubicin-treated human and mouse fibroblasts resulted in a striking increase in spheroid size compared with incubation with conditioned medium from control fibroblasts (Figure 4C). To confirm that the increased growth of the spheroids resulted from increased tumor cell proliferation as opposed to decreased cell death, two approaches were taken. First, tumor spheroids were fixed and embedded, and sections were stained for the proliferation marker Ki67 (Figure 4D). As the experiments were conducted in low (2%) serum conditions, few, if any, Ki67^+^ cells were detected in spheroids cultured with control-conditioned medium. By contrast, spheroids incubated with chemotherapy-treated fibroblast-conditioned medium were larger in size and had a ring of Ki67^+^ cells around their periphery. Second, tumor cell colony formation assays showed no difference in colony number in the presence of chemotherapy-treated fibroblast-conditioned medium, but a significant increase in colony size (3T3 conditioned medium) and an increased trend toward MRC-5-conditioned medium (Figure 4E). To investigate the mechanism underlying this increased 3D tumor spheroid proliferation, D2A1 mouse or ZR-75-1 human tumor spheroids were treated with conditioned medium for 30 min, lysed and then assayed by phosphoprotein array. Increased phosphorylation of several key mitogenic factors was observed in the spheroids incubated with doxorubicin-treated fibroblast-conditioned medium, in particular increased phosphorylation of Tyr701 STAT1, Tyr705 STAT3, and pan-SRC in the D2A1 spheroids and increased phosphorylation of Ser473 AKT, Thr202/Tyr204 ERK p42/p44, and Tyr705 STAT3 in the ZR-75-1 spheroids (Figure 4F). These findings are consistent with the activation of STAT3 signaling downstream of SASP cytokines such as IL-6 and LIF,^25^^,^^26^^,^^50^ whose expression is upregulated in chemotherapy-treated fibroblasts, promoting proliferation of mouse and human tumor cells in both *in vitro* and *in vivo* models.

### Chemotherapy-treated fibroblasts can be selectively eliminated *in vitro*

**Figure 5:**
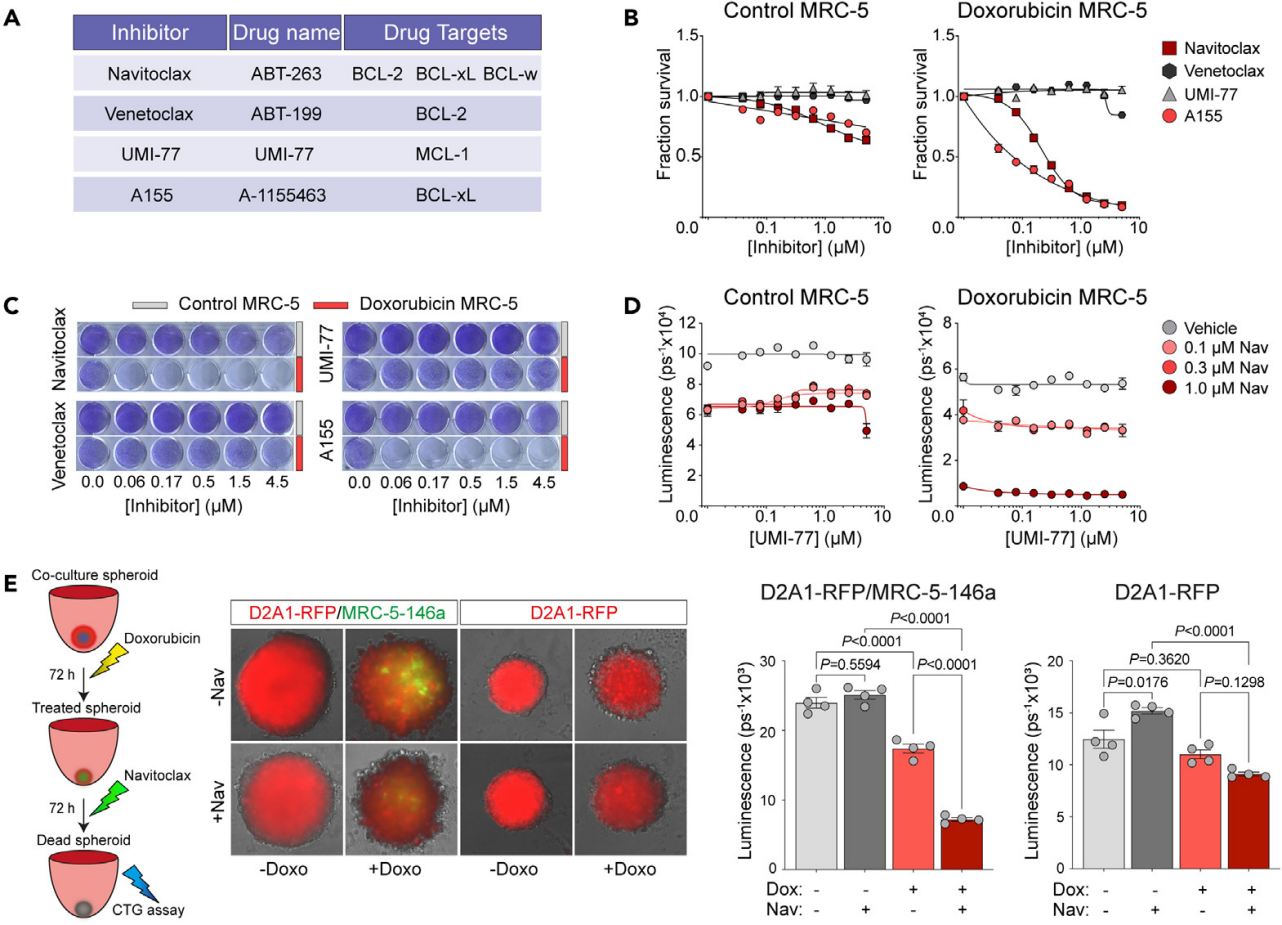
Navitoclax eliminates chemotherapy-induced senescent fibroblasts *in vitro* (A) Details of the BCL-2 family inhibitors used in the study. (B) MRC-5-146a fibroblasts were treated with 1.7 μM doxorubicin for 24 h. Nine days after treatment withdrawal, control or doxorubicin-treated fibroblasts were plated into 96-well plates (see STAR Methods; n = 3 wells per condition). After 24 h, fibroblasts were treated with inhibitors as indicated and incubated for 72 h before cell viability was measured by CellTiter-Glo. (C) Doxorubicin-treated MRC-5 fibroblasts, 10 days following treatment withdrawal, or control fibroblasts were seeded into 24-well plates (see STAR Methods; n = 1 well per condition), then 24 h later treated inhibitors as indicated. After 72 h, plates were fixed and stained with crystal violet. (D) Doxorubicin-treated MRC-5 fibroblasts, 9 days following treatment withdrawal, or control fibroblasts were plated into 96-well plates (n = 3 wells per condition). After 24 h, fibroblasts were treated with a range of concentrations of UMI-77, alone or in combination with 3 concentrations of navitoclax, and incubated for 72 h before cell viability was measured by CellTiter-Glo assay. (E) 5,000 D2A1-RFP mouse tumor cells were seeded alone or co-seeded into ultra-low attachment U-bottom 96-well plates with 5,000 MRC-5-146a human fibroblasts (n = 4 wells per condition). After 24 h, spheroids were treated with 170 nM doxorubicin or vehicle for 72 h, followed by treatment with 4.5 μM navitoclax or vehicle for a further 72 h. At the end of the assays spheroids were imaged (scale bar, 100 μm), and cell viability was quantified by CellTiter-Glo (one-way ANOVA). (B–E) Equivalent results were obtained in 2–4 independent experiments.

Prolonged survival of senescent cells requires the avoidance of apoptotic-mediated cell death, commonly by upregulating expression of anti-apoptotic BCL-2 family members.^41^^,^^51^ BCL-2 family proteins bind to and inhibit BH3-initiator proteins such as BAD, which would otherwise activate the apoptotic effector protein BAX to trigger caspase activation and mitochondrial outer membrane permeabilization.^52^ Consequently, agents which target the BCL-2 family members, such as BCL-2, BCL-xL, BCL-w, and MCL-1 (Figure 5A), have been assessed for their efficacy in triggering apoptosis. In particular, navitoclax, which targets BCL-2, BCL-xL, and BCL-w, has been shown to selectively kill senescent cells *in vivo*^53^^,^^54^^,^^55^^,^^56^^,^^57^ and has been evaluated in human clinical trials for aging-/senescence-related indications. However, there has been little focus on their use in targeting therapy-induced senescent fibroblasts.

Fibroblasts treated with doxorubicin for 24 h and then incubated for 10 days in the absence of drug were effectively killed by treatment with either navitoclax or the BCL-xL-specific inhibitor A-1155463 (A115), whereas these inhibitors had limited effect on control vehicle-treated fibroblasts (Figure 5B). By contrast, treatment with either the BCL-2-only inhibitor venetoclax or the MCL-1 inhibitor UMI-77 had no impact on chemotherapy-treated or control fibroblast viability. Comparable results were obtained using human IMR-90 fibroblasts, mouse 3T3 fibroblasts, and fibroblasts treated with docetaxel (Figures S3A–S3D). A concern in these assays is that CellTiter-Glo monitors ATP levels in the cell, and it is likely that non-proliferating cells have different basal ATP metabolism. Equivalent results were obtained using non-enzymatic crystal violet staining (Figures 5C and S3A). As MCL-1 exhibits some functional redundancy with other BLC2 family members,^58^ the effect of combining the MCL-1 inhibitor UMI-77 with either navitoclax (Figures 5D and S3A) or A115 (Figure S3D) was examined. In combination, the addition of an MCL-1 inhibitor did not enhance killing of chemotherapy-treated fibroblasts *in vitro*. Finally, equivalent results were obtained treating fibroblasts with navitoclax formulated in corn oil (not shown), Phosal, or culture medium (Figures S3B and S3E).

To assess the ability of navitoclax to target chemotherapy-treated fibroblasts in a more complex environment, RFP-labeled D2A1 tumor cells alone or together with MRC-5-miR146a-GFP fibroblast were cultured as 3D spheroids and treated for 72 h with vehicle or doxorubicin and then for a further 72 h in the presence or absence of navitoclax (Figure 5E). As previously observed (Figure 3D), doxorubicin reduced viability of D2A1 cells *in vitro*, illustrated by the reduction in RFP expression within the spheroids. This effect was more evident in the co-culture spheroids and accompanied by a concomitant increase in expression of the fibroblast GFP senescence reporter. Subsequent navitoclax treatment had no effect on tumor-cell-only spheroid viability and similarly no impact on vehicle-treated co-culture 3D spheroids. In contrast, in chemotherapy-treated co-culture spheroids, navitoclax treatment resulted in a further significant reduction in cell viability and a notable loss of GFP-expressing fibroblasts (Figure 5E).

### Combination chemotherapy and navitoclax treatment *in vivo*

**Figure 6:**
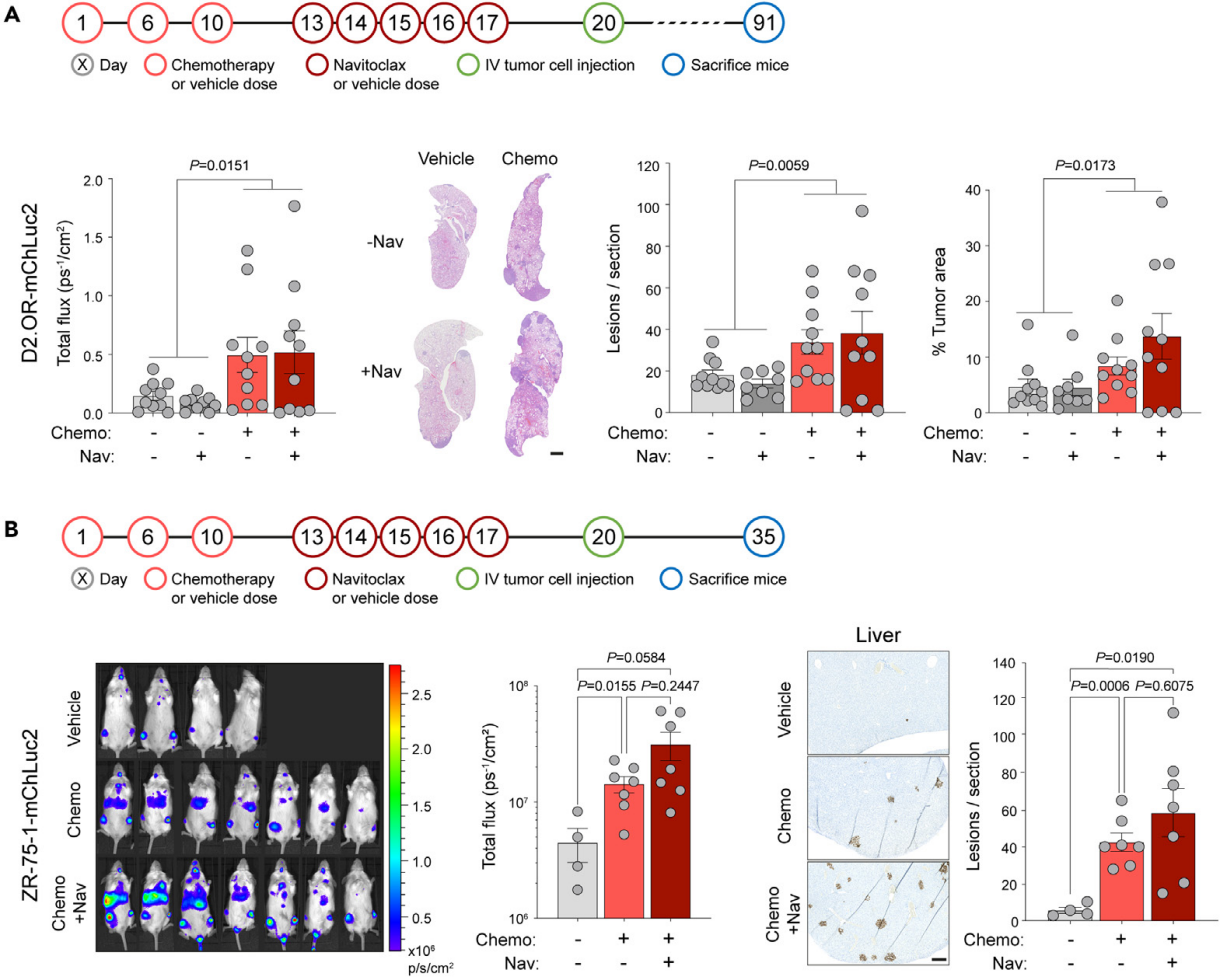
Effect of combined chemotherapy and navitoclax treatment on metastasis and survival (A) Schedule for treatment of BALB/c mice with chemotherapy (Chemo) or vehicle followed by navitoclax (Nav) or vehicle, and subsequent intravenous (IV) inoculation of 1 × 10^6^ D2.OR-mChLuc2 tumor cells (n = 9–10 mice per group). Quantification of *in vivo* IVIS imaging of the thoracic region on day 77 (mean ± SEM, Mann-Whitney *U*-test between vehicle and chemotherapy groups combined). Quantification of lung metastatic burden at termination of the experiment (day 91). Representative H&E lung sections (scale bar, 1 mm). Number of metastatic lesions per lung section. Percentage tumor burden in the lungs (mean ± SEM, Mann-Whitney *U*-tests between vehicle and chemotherapy groups combined). (B) Schedule for treatment of NOD Rag gamma mice and subsequent IV inoculation of 2 × 10^6^ ZR-75-1-mChLuc2 tumor cells. *In vivo* IVIS quantification of mice (n = 4 mice vehicle group, n = 7 mice Chemo and Chemo + Nav groups) on day 34 (±SEM, Welch’s ANOVA). Representative lamin A/C staining in the liver (scale bar, 250 μm) and quantification of metastatic lesions in the liver (mean ± SEM, Welch’s ANOVA.

Encouraged by the efficacy of navitoclax *in vitro*, we investigated whether these agents could reduce the impact of prior chemotherapy treatment on metastatic tumor outgrowth. BALB/c mice were treated with a course of chemotherapy or vehicle followed by 5 daily doses of navitoclax or vehicle and 3 days later inoculated intravenously with D2.OR tumor cells (Figure 6A). Consistent with previous data (Figures 1A and 1B), on day 77, there was significantly increased tumor burden in the chemotherapy-treated mice compared with vehicle-treated mice, monitored by IVIS imaging, however, no significant difference between groups which had, or had not, received subsequent navitoclax treatment. On day 91, when the first mice began to show signs of ill health, the experiment was terminated. In line with the day 77 IVIS data, chemotherapy-treated mice had a significantly increased metastatic burden in the lungs, quantified by the numbers of metastatic lesions and percentage tumor area. Again, no significant effect of navitoclax treatment on vehicle-treated mice was observed. Equivalent results were obtained in NRG mice inoculated with human ZR-75-1 breast cancer cells (Figure 6B). As reported here (Figure 1E), mice pre-treated with a course of chemotherapy develop significantly more ZR-75-1 liver metastases than vehicle-treated mice (Figure 6B) but subsequent treatment with navitoclax did not reduce metastatic outgrowth.

**Figure 7:**
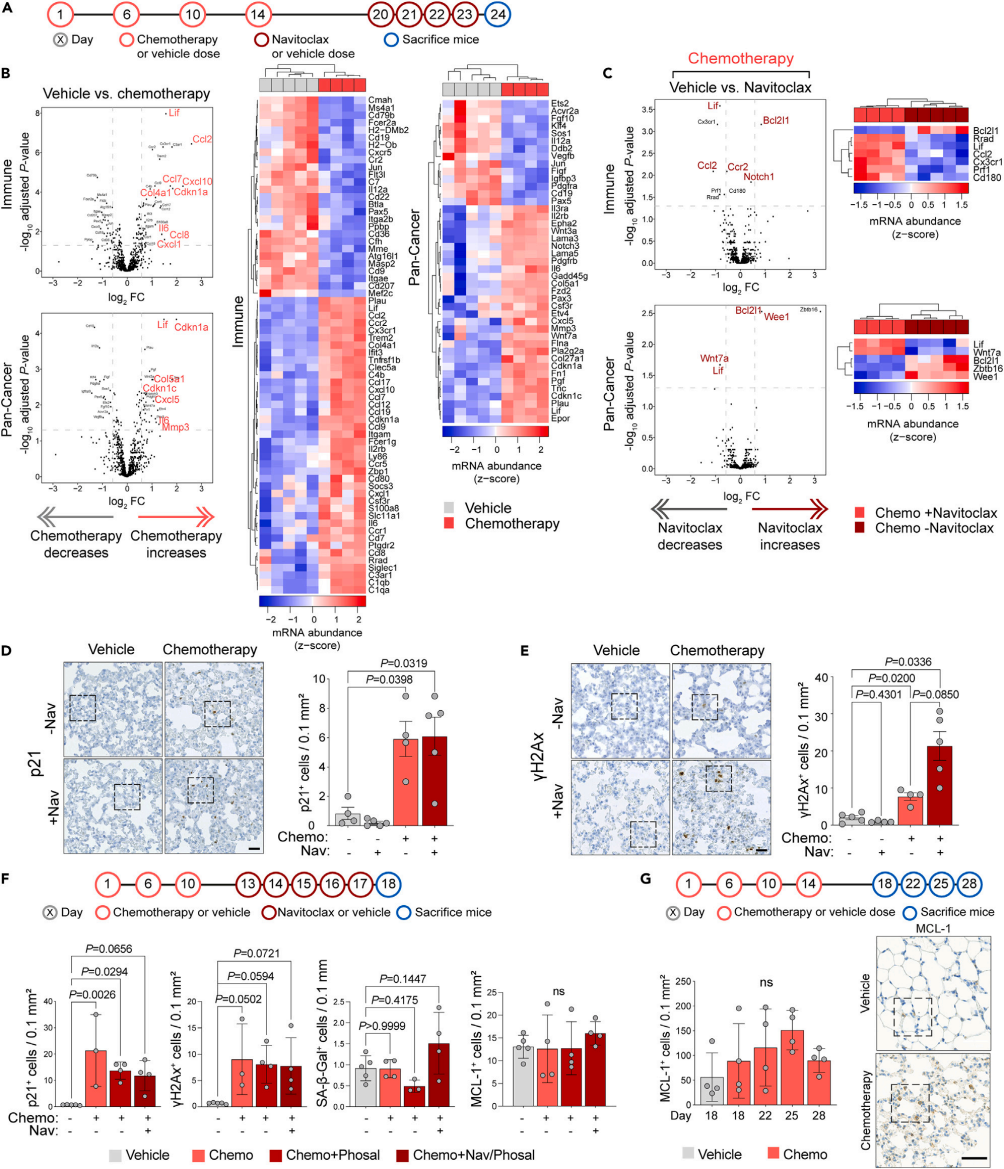
Navitoclax does not reverse chemotherapy-induced tissue damage *in vivo* (A) Schedule for the treatment of BALB/c mice with chemotherapy (Chemo) or vehicle followed by navitoclax (Nav) or vehicle. (B and C) Mice were sacrificed on day 24; RNA was extracted from snap-frozen lung tissue and gene expression profiling performed using the NanoString mouse PanCancer Immune and PanCancer Pathways panels. Normalization was carried out as described in Figure 2B. Volcano plots showing differentially expressed genes between chemotherapy- and vehicle-treated mice (B) or between chemotherapy-treated mice subsequently treated with navitoclax or vehicle (C). Genes with absolute log_2_ fold change ≥0.585 and FDR adjusted p value <0.05 were considered significant and shown in the associated heat maps. Genes highlighted in red are discussed in the text. (D and E) Samples from (B) and (C) were stained for γH2Ax and p21 (Dako) (scale bars, 25 μm) and quantified (Welch’s ANOVA). Higher power images are shown in Figures S5B and S5C. (F) Schedule for the treatment of BALB/c mice with chemotherapy or vehicle, followed by navitoclax or vehicle (Phosal). Mice were sacrificed one day after the final dose of navitoclax. Lung tissue was divided, prepared either for FFPE or snap-frozen embedded in OCT, and sections were stained for p21 (Abcam), γH2Ax, SA-β-Gal, or MCL-1 and quantified (mean ± SD, one-way ANOVA). (G) Schedule for the treatment of BALB/c mice with chemotherapy or vehicle. Mice were sacrificed 4, 8, 11, and 14 days after the final dose of chemotherapy. Lung sections were stained for MCL-1. Quantification of MCL-1 staining on indicated days (mean ± SD, one-way ANOVA). Representative images (day 18, vehicle; day 22, chemotherapy; scale bar, 50 μm). Higher power images of indicated areas are shown in Figure S5D.

To investigate this lack of efficacy in limiting metastatic outgrowth, naive BALB/c mice were treated with a course of vehicle or chemotherapy followed by 4 daily doses of vehicle or navitoclax, and 24 h after the last dose (day 24) lungs were profiled using the NanoString PanCancer Immune and Pathways panels (Figure 7A). Lungs isolated from mice receiving chemotherapy alone showed, as previously reported (Figure 2B), a significantly upregulated SASP expression, including increased expression of *Il6*, *Lif*, *Cdkn1a* (p21), *Mmp3*, *Ccl2*, *Ccl7*, *Cxcl1*, and *Cxcl10* (Figure 7B; Tables S1 and S2), whereas PCA analysis revealed discrete clustering of the chemotherapy and vehicle groups (Figure S4A). Of note, when analyzing these data together with the data shown in Figure 2B, the vehicle and chemotherapy-treated samples from the independent experiments clustered together (Figure S4B), indicating a reproducible effect of chemotherapy treatment on the lung tissue despite having different experimental endpoints (Tables S1 and S2). Similarly, assessment of immune cell abundance scores again showed a decrease in B cells and significant increase in dendritic cells (Figure S5A).

Lung tissue from mice treated with navitoclax as a monotherapy showed no significant differentially expressed genes in either of the NanoString panels. In addition, consistent with the lack of efficacy in metastasis assays, there was little indication that navitoclax reverses the effects of chemotherapy treatment, with only 10 genes within the two panels showing a significant change in expression in mice receiving a subsequent course of navitoclax (Figure 7C) and no impact on the abundance of any immune cell populations (Figure S5A). Importantly, within these chemotherapy-treated groups, although subsequent navitoclax administration significantly reduces the expression of *Lif* and *Ccl2* as well as the CCL2 receptor *Ccr2*, there was no significant reduction in expression of p21 (*Cdkn1a*). To confirm these findings, lung sections from the same mice were stained for p21. As previously reported (Figure 2D), few p21^+^ cells are detected in the lung tissue of vehicle-treated mice but their number significantly increased in lungs of chemotherapy-treated mice (Figures 7D and S5B). In chemotherapy-treated mice that received a subsequent course of navitoclax, there was no change in the number of p21^+^ cells (Figure 7D), confirming that, and in contrast to the *in vitro* findings (Figure 5), navitoclax is unable to eliminate p21^+^ cells *in vivo*. Moreover, γH2Ax^+^ cells are readily detected in chemotherapy-treated mice (Figures 7E and S5C). Similar results were obtained in an independent experiment using different formulation of navitoclax delivery system (see STAR Methods) and an alternative p21 antibody (Figure 7F). Finally, navitoclax treatment failed to deplete SA-β-Gal^+^ cells regardless of the formulation strategy. Although chemotherapy-treated fibroblasts showed no sensitivity to MCL-1 inhibition *in vitro* (Figures 5B and S3A–S3D), it remained a possibility, given its functional redundancy with other BLC-2 family members,^58^ that upregulation of MCL-1 *in vivo* might promote escape from navitoclax-mediated killing. However, there was no significant change in MCL-1^+^ cell number following chemotherapy treatment, with or without a subsequent course of navitoclax (Figures 7F, 7G, and S5D). By contrast, expression of *Bcl2l1*, which encodes BCL-xL, was significantly upregulated in the chemotherapy plus navitoclax group, raising the prospect that upregulation of BCL-xL could provide an *in vivo* resistance mechanism allowing DNA-damaged senescent stromal cells to survive navitoclax treatment.

## Discussion

Here, we show that in multiple independent models of both immune-competent and immune-compromised mice, pre-treatment with a clinically relevant chemotherapy regimen of combined doxorubicin and cyclophosphamide followed by subsequent inoculation of tumor cells results in a significantly increased burden of metastatic disease. The use of the mouse D2.OR and human ER^+^ ZR-75-1 tumor cell lines, which, when inoculated into naive mice, give rise to dormant DTCs at secondary sites, has clinical relevance in the context of ER^+^ breast cancers, which frequently exhibit a late relapse phenotype developing metastatic disease years or decades after original diagnosis and treatment.^59^^,^^60^^,^^61^^,^^62^ In both the mouse models and patients, DTCs have a prolonged exposure to the stromal microenvironment, which would be rendered a more pro-metastatic environment in patients receiving systemic chemotherapy as part of their treatment. In addition, although a late relapse phenotype is much less common in hormone-receptor-negative breast cancers and these cancers frequently respond well to chemotherapy, there remains a significant proportion of patients with chemoresistant residual disease. Consequently, in both ER^+^ and ER^−^ scenarios, the data presented here highlight the potential negative consequences of chemotherapy treatment in which chemotherapy-mediated damage in normal tissue, paradoxically, has the potential to increase the risk of recurrence.

Here, we demonstrate that systemic delivery of chemotherapy causes DNA damage in normal mouse tissues and increased accumulation of cells expressing SASP factors and staining positive for the senescence markers p21 and SA-β-Gal. The clinical importance of targeting both naturally occurring and therapy-induced senescent cells has become increasingly clear.^63^^,^^64^ Seminal proof of principle experiments, involving the genetic elimination of cells expressing the senescence marker p16^Ink4a^, has demonstrated an alleviation of age- and therapy-induced pathologies,^5^^,^^6^ including progression and metastasis in a PyMT-MMTV spontaneous mammary carcinoma model.^37^ Experimental studies into the therapeutic targeting of senescent cells has focused on administration of the BLC-2/BCL-xL/BCL-w inhibitor navitoclax (ABT-263), the BCL-2/BCL-xL inhibitor ABT-737, or the non-related combination of dasatinib and quercetin (D&Q), which have been shown to eliminate or reduce the number of senescent cells in aging mouse models and alleviative the aging phenotype.^53^^,^^65^^,^^66^

In our studies, D&Q was not selective for senescent fibroblasts *in vitro* and had no effect *in vivo* (data not shown). By contrast, navitoclax or the BCL-xL-specific inhibitor A115 selectively eliminated chemotherapy-induced senescent fibroblasts *in vitro* and impaired their ability to promote tumor cell proliferation. However, using dosing regimens shown to be effective in aging mouse models,^53^ navitoclax treatment failed to eliminate chemotherapy-induced p21^+^ cells *in vivo* and thus did not reduce metastatic outgrowth in different chemotherapy-treated mouse models. Although gene expression profiling provided some evidence of on-target effects of navitoclax in chemotherapy-treated mice, the number and magnitude of the changes were small. As the number of SA-β-Gal^+^ cells was substantially lower than the number of p21^+^ cells (Figures 2D and 7F), this likely reflects that a course of systemic chemotherapy treatment results in the accumulation of damaged SASP-producing fibroblasts and other stromal cells sufficient to create a pro-tumorigenic metastatic niche but insufficient to induce a BCL-xL-dependent phenotype. In addition, mice receiving the combination treatment showed an increased expression of *Bcl2l1* (encoding the navitoclax target BCL-xL), raising the possibility that, as has been observed in models of non-Hodgkin lymphoma,^67^ increased BCL-xL levels may contribute to BCL-2/BCL-xL inhibitor resistance. Of note, MCL-1 exhibits some functional redundancy with other BLC-2 family members^58^; however, chemotherapy treatment did not significantly increase the number of MCL-1^+^ cells in the lung.

Although our 3D *in vitro* models support the hypothesis that increased SASP signaling by stromal fibroblasts directly promotes tumor growth by increasing mitogenic signaling and tumor cell proliferation; there are other consequences that chemotherapy-exposed cells could inflict on the microenvironment, contributing indirectly to metastatic colonization. For example, the SASP can exhibit immune suppressive properties via the promotion of type-2 immune polarization,^68^ resulting in reduced immune surveillance. Indeed, our cytokine array data reveal that among the most abundant chemokines secreted by chemotherapy-treated fibroblasts are macrophage inflammatory protein 1γ (MIP-1γ, CCL-9) and macrophage chemoattractant protein 1 (MCP-1, CCL-2), which is reflected in increased expression of *Ccl9* and *Ccl2* in the chemotherapy-treated lung tissue. Interestingly, NanoString profiling of chemotherapy-treated lungs revealed a decrease in B cells and increase in dendritic cells. However, a profound pro-tumorigenic effect of chemotherapy treatment is observed in NRG mice which lack a functional immune system, including having defective dendritic cells. By contrast, notable in our studies was the increased expression of extracellular matrix remodeling and fibrosis genes in chemotherapy-treated lungs, features associated with aging tissues and increased metastatic outgrowth in multiple cancer models.^2^^,^^46^^,^^47^

In summary, this study highlights the role of the metastatic microenvironment in controlling outgrowth of disseminated tumor cells and the need to identify additional therapeutic approaches to effectively limit chemotherapy-induced normal tissue damage and its pro-tumorigenic effects.

### Limitations of the study

This study is limited by not investigating the spectrum of stromal cell types that become senescent, and those that show an SASP-positive phenotype but are not senescent, and their persistence over time. To address this would require a full time course experiment coupled with single-cell sequencing analysis or in-depth multiplex immunohistochemical approaches. The second main limitation is the failure to recapitulate the *in vitro* studies demonstrating that chemotherapy-treated fibroblasts can be eliminated by inhibition of BCL-xL in *in vivo* models, despite using two different formations of the navitoclax (corn oil and Phosal-based formations) which have been used effectively in other *in vivo* studies (corn oil^69^^,^^70^^,^^71^; Phosal^53^^,^^54^^,^^55^^,^^56^). Further experiments will be required to test our hypothesis that this reflects (1) the limited ability of a course of systemic chemotherapy to drive BCL-xL-dependent senescence *in vivo* and/or (2) that chemotherapy-treated cells *in vivo* upregulated alternative pro-survival pathways.

## STAR★Methods

### Key resources table

**Table:** 

REAGENT or RESOURCE	SOURCE	IDENTIFIER
**Antibodies**		

p21	Dako	Cat#M7202; RRID:AB_2077700
p21	Abcam	Cat#ab188224; RRID:AB_2734729
γH2Ax (S139)	Cell Signaling	Cat#9718S; RRID:AB_2118009
α Smooth muscle actin	Sigma	Cat#A2547; RRID:AB_2764436
EpCAM	Abcam	Cat#ab221552; RRID:AB_3065022
Endomucin (V7C7)	Santa Cruz	Cat#sc65495; RRID:AB_2100037
CD4	Ebioscience	Cat#14-9766: RRID:AB_2573007
CD8	Ebioscience	Cat#14-0808; RRID:AB_2572771
Ki67	Abcam	Cat#ab16667; RRID:AB_302459
MCL-1	Cell Signaling	Cat#94296; RRID:AB_2722740
Firefly luciferase	Abcam	Cat#ab181640; RRID:AB_2889835
Lamin A/C	Abcam	Cat#ab108595; RRID:AB_10866185
Mouse IgG-488	Invitrogen	Cat#A11001; RRID:AB_2534069
Mouse IgG-555	Invitrogen	Cat#A21127; RRID:AB_2535769
Rat IgG-488	Invitrogen	Cat#A11006; RRID:AB_2534074
Rabbit IgG-488	Invitrogen	Cat#A11008; RRID:AB_143165

**Bacterial and virus strains**		

Lucifierase (firefly) lentiviral particles, firefly luciferase gene with a blasticidin-resistance gene	Amsbio	Cat#LVP326
PGK-H2BmCherry-IRIS-Luc2	Genscript	N/A
Lentiviral vector pDEST/pHIV-H2BmRFP-rfa_verB, RFP encoding gene	Gift from Matthew Smalley, University of Cardiff)	N/A
PmiR146a-GFP plasmid	Gift from Steve Elledge laboratory	Kang et al.^48^

**Chemicals, peptides, and recombinant proteins**		

Navitoclax	Selleckchem	Cat#S1001
Venetoclax	Selleckchem	Cat#S8048
A-1155463	Selleckchem	Cat#S7800
UMI-77	Selleckchem	Cat#S7531
Doxorubicin	Selleckchem	Cat#S1208
Docetaxel	Selleckchem	Cat#S1148
Cyclophosphamide monohydrate	Sigma	Cat#C0768

**Critical commercial assays**		

Senescence cells histochemical staining kit	Sigma	Cat#G6257
SA-β-Gal histochemical staining kit	Sigma	Cat#CS0030
NanoString mouse PanCancer Immune panel	NanoString	N/A
NanoString mouse PanCancer Pathways panel	NanoString	N/A
Cytokine arrays	RayBiotech	Cat#G2000
Phosphoprotein arrays. Pathscan antibody-array slides	Cell Signalling Technologies	Cat#7982
CellTiter-Glo assay	Promega	Cat#G7570
Stem Elite ID System	Promega	Cat#G9530

**Deposited data**		

NanoString data for Figures 2B, 7B, and 7C	This paper	Zenodo; https://doi.org/10.5281/zenodo.10045006

**Experimental models: Cell lines**		

MRC-5	ATCC	Cat#CCL-171
IMR-90	ATCC	Cat#CCL-186
NIH-3T3	Laboratory stocks	-
ZR-75-1	Laboratory stocks	STR tested every 4 months
D2A1	Ann Chambers laboratory	Morris et al.^23^
D2.OR	Ann Chambers laboratory	Morris et al.^23^
D2A1-m2	Generated in our laboratory	Jungwirth et al.^72^

**Experimental models: Organisms/strains**		

BALB/c (BALB/cAnNCrl) mice, female, 6 -9 weeks of age	Charles River	Cat#028
NOD RAG gamma (NRG) mice (NOD-Rag1^-/-^ IL2rg^-/-^), female, 6 -9 weeks of age	Charles River	Cat#IMSR_JAX:007799

**Oligonucleotides**		

Taqman gene expression assays, see Table S3		

**Software and algorithms**		

R package NanoStringNorm	-	v1.2.1
R package limma	-	v3.40.6
R package FactoMineR	-	v2.3
R statistical programming language	-	v3.6.14
GraphPad Prism	-	v8 or v9
QuPath	-	v0.3.2 Bankhead et al.^49^
Living Image software	PerkinElmer	v4.5

**Other**		

17β-oestradiol pellets, sustained release 0.36 mg	Innovative Research of America	Cat#NE-121
D-Luciferin Firefly, potassium salt, 1.0 g /vial	Caliper Life Sciences	Cat#119222

### Resource availability

#### Lead contact

Further information and requests for resources and reagents should be directed to and will be fulfilled by the lead contact, Clare Isacke (clare.isacke@icr.ac.uk).

#### Materials availability

All unique/stable reagents generated in this study are available from the lead contact upon request.

#### Data and code availability

• The NanoString data presented in Figures 2B, 7B, and 7C have been deposited at Zenodo and are publicly available. The accession number is listed in the key resources table.
• This paper does not report original code.
• Any additional information required to reanalyze thee data reported in this paper is available from the lead contact upon request.
• All other raw data are available from the lead contact upon request.

### Experimental model and study participant details

#### Animals

All animal work was carried out under UK Home Office Project Licenses 70/7413 and P6AB1448A granted under the Animals (Scientific Procedures) Act 1986 (Establishment Licence, X702B0E74 70/2902) and was approved by the 'Animal Welfare and Ethical Review Body' at The Institute of Cancer Research (ICR). Mice were purchased from Charles River. All mice used were female and aged between 6-9 weeks and 18-25 g in weight at the beginning of an experiment. Syngeneic studies were carried out in BALB/c mice. Human cell line studies were carried out in NOD RAG gamma (NRG) mice (NOD-Rag1^-/-^ IL2rg^-/-^), implanted with 90 day, sustained release 0.36 mg 17β-oestradiol pellets (Innovative Research of America, NE-121) 4-5 days prior to tumor cell implantation. All mice were group housed in individually ventilated cages and kept at 21°C ± 2°C, humidity level between 45-65% and light/dark cycle of 12 hours. Mice were monitored daily by ICR Biological Services Unit staff and had food and water *ad libitum*. In all cases, experiments were terminated if the primary tumor reached a maximum allowable diameter of 17 mm, if thoracic IVIS signal exceeded 1 x 10^9^ photons per second or if a mouse showed signs of ill health. For non-tumor bearing mice, mice were randomized into groups based on body weight. For experimental and spontaneous metastasis experiments, mice were randomized prior to drug administration based on IVIS signal or tumor volume, respectively. Dosing schedules for individual experiments are presented in the figures.

#### Cells

MRC-5 and IMR-90 fibroblasts were purchased from ATCC in 2009 and 2016, respectively. NIH-3T3 and ZR-75-1 cells were from Isacke laboratory stocks. D2A1 and D2.OR cells were from Ann Chambers’ laboratory stocks.^23^ The generation of the metastatic D2A1-m2 subline has been described previously.^72^ All cells were used within ten passages after resuscitation and were routinely subjected to mycoplasma testing. ZR-75-1 cells were short tandem repeat tested every 4 months (Stem Elite ID System, Promega). Unless otherwise stated, all cells were cultured in DMEM (ThermoFisher) supplemented with 10% FBS (ThermoFisher), 100 μg/mL benzylpenicillin sodium & 60 μg/mL streptomycin sulphate, in humidified incubators at 37°C and 5-10% CO_2_. Cells were grown to 80-90% confluency and passaged at the desired fraction every 2-4 days.

### Method details

#### Reagents

Doxorubicin (S1208), docetaxel (S1148), navitoclax (S1001), A-1155463 (S7800), venetoclax (S8048) and UMI-77(S7531) were purchased from Selleckchem. Cyclophosphamide monohydrate was purchased from Sigma-Aldrich (C0768). RTqPCR probes are listed in the key resources table with further detail in Table S3. Antibodies are listed in the key resources table with the dilutions used detailed in Table S4.

#### Transfection of cells

As indicated, tumor cells were transduced with lentiviral expression particles containing either: (1) a firefly luciferase gene with a blasticidin-resistance gene (cells denoted -Luc) (Amsbio, LVP326); (2) a firefly luciferase gene, with an mCherry encoding gene (cells denoted -mChLuc2) (PGK-H2BmCherry-IRIS-Luc2; Genscript); or (3) an RFP encoding gene (cells denoted -RFP) (lentiviral vector pDEST/pHIV-H2BmRFP-rfa_verB, a gift from Matthew Smalley, University of Cardiff). mCherry^+^/RFP^+^ cells were selected by fluorescence-activated cell sorting (FACS). Luciferase only transduced cells were enriched by culturing the cells in DMEM with 10% FBS containing blasticidin for 2-3 passages. For transfection of fibroblasts with PmiR146a-GFP plasmid, the miR146a-GFP plasmid (35 μg)^73^ was combined with packaging vectors pMD2.G (11 μg) and psPAX2 (25.6 μg) in 18 mL Optimem medium containing 216 μL Lipofectamine-2000, and incubated at room temperature for 20 minutes. Lentivirus containing medium from HEK293T cells was harvested at 24 and 48 hours, centrifuged at 300*g* and filtered through a 0.2 μm syringe filter before use for cell transfections. Fibroblasts were seeded in 25 cm^2^ flasks in medium containing miR146a-GFP lentiviral particles and 4 μg mL^-1^ polybrene and for incubated 24 hours. Virus containing medium was replenished after 24 hours. Transfected fibroblasts were then maintained in DMEM with 10% FBS containing puromycin for 2-3 passages.

#### Chemotherapy and navitoclax treatment of mice

Mice were dosed via 100 μL intraperitoneal injection with a combination of doxorubicin (2.3-2.7 mg kg^-1^, Selleckchem S1208) and cyclophosphamide (40-44 mg kg^-1^, Sigma 50850) in 0.9% NaCl. Doxorubicin and cyclophosphamide were stored separately at a 2X concentration (1.7 mM and 57 mM, respectively) and were mixed at a 1:1 ratio immediately prior to injection. Vehicle-treated mice were injected intraperitoneally with 100 μL 0.9% NaCl. Navitoclax was stored as aliquots of 100 mM solution in DMSO at -80°C and was administered as a dose of 50 mg/kg daily dose. For each dose one aliquot was thawed and diluted into either corn oil to a final concentration of 5 mM (5% DMSO final concentration) or, where indicated, in 10% ethanol:30% PEG 400 (Sigma 202398):60% Phosal 50 PG (LIPOID, LLC 368315). Vehicle-treated mice were treated with a 5% solution of DMSO with corn oil or the Phosal mixture. Mice were dosed via oral gavage daily for 5 days using a 200 μL volume.

#### Tumor cell inoculation into mice

For Intravenous inoculation, 5 x 10^5^ D2A1, 1 x 10^6^ D2.OR or 2 x 10^6^ ZR-75-1 cells (transfected with either -Luc or -mChLuc2 vectors) were injected into the lateral tail vein of mice. Metastatic growth was monitored by repeated IVIS imaging starting at ∼90 minutes following inoculation. At endpoint lungs were excised and, where indicated, IVIS imaged *ex vivo*. For spontaneous metastasis assays, 2 x 10^5^ D2A1-m2 cells were injected orthotopically into the 4^th^ mammary fat pad under general anesthesia. Tumor growth was measured twice a week using calipers up to a maximum diameter of 17 mm. Tumor volume was calculated as 0.5236 x [(width + length)/2]ˆ3. At endpoint tumors were excised and weighed.

#### IVIS imaging of mice

Mice were injected intraperitoneally with 150 mg kg^-1^ D-luciferin (Caliper Life Sciences) in 100 μL and mice imaged *in vivo* using an IVIS imaging chamber (IVIS Illumina II). Luminescence measurements (photons per second per cm^2^) were acquired over 1 minute and analyzed using the Living Image software (PerkinElmer) using a constant size region of interest over the tissues. Alternatively, >15 minutes after D-luciferin injection, dissected lungs and/or livers were imaged *ex vivo*.

#### Quantification of metastatic burden and immunostaining

Tissues were formalin-fixed and paraffin-embedded (FFPE). 3-4 μm lung, liver or bone FFPE sections were cut, dewaxed in xylene, re-hydrated through ethanol washes and stained with hematoxylin and eosin (H&E). Slides were scanned using Hamamatsu microscope with a NanoZoomer XR camera using the 20x objective and file names blinded. For quantifying tumor burden, total number of individual nodules was counted manually in 1-3 sections approximately 150 μm apart, per tissue. Lung metastatic area was quantified as the mean size of the nodules or percentage of the area of the metastatic nodules normalized to the total lung area. Alternatively, sections were subjected to high-temperature antigen retrieval, blocked using avidin/biotin, before incubation with primary antibodies. Immunohistochemical detection was achieved with the VectaStain ABC system. Stained sections were scanned as described above and file names blinded. p21, γH2Ax or MCL-1 positive cells were quantified either manually from ≥6 randomly selected 0.1 mm^2^ fields of view per lung section, or by automated cell detection of whole tissue sections using QuPath,^49^ avoiding regions containing bronchi and bronchioles. Alternatively, FFPE sections of mouse lungs were stained with p21 alongside αSMA, endomucin or EpCAM and DAPI, then incubated with the appropriate secondary antibody conjugated to fluorophores. Fluorescent stained sections were scanned as described above. p21 positive cells were counted across the whole section. p21 positive cells were then scored for positivity of αSMA, endomucin or EpCAM. Representative higher power images were collected on a Leica SP8 microscope using a 40X oil immersion objective.

#### Senescence-associated β-galactosidase staining of lung tissue sections (SA-β-Gal)

Lungs were excised, lobes dissected, and left lobe was frozen in OCT embedding matrix (CellPath KMA-0100-00A) on dry ice. Frozen blocks were sectioned in cryostat to 10 μm thick sections. Sections were air-dried before fixing for 10 minutes in 2% formaldehyde (Sigma F8775) : 0.2% glutaraldehyde (Sigma G6257) solution in PBS. Slides were washed three times with PBS and incubated with X-gal staining mixture (Sigma CS0030) at 37°C without CO_2_ overnight. Next day, the slides were washed in PBS and counterstained with hematoxylin (Sigma MHS1) before mounting.

#### *Ex vivo* lung culture

Following sacrifice, mouse lungs were perfused with agarose, removed and allowed to solidify in cold PBS for ∼1 hour. Lungs were cut into 150 μm slices and cultured on an inert matrix in M199 medium for 24 hours before seeding of tumor cells.

#### RNA extraction from tissue

Mice were sacrificed 7 or 10 days after the final chemotherapy or vehicle treatment. The lungs were excised and the left lobe was fixed and processed for immunohistochemical staining as described above. The right lobes were immediately snap-frozen in liquid nitrogen in a cryovial and then stored at -80°C. To extract RNA, a ∼20 mg chunk of frozen lung tissue was collected using a scalpel and placed in 1 mL RLT buffer (Qiagen) with 1:100 β-mercaptoethanol in a Precellys silicone bead dissociation tube (Bertin-Corp). Tissue were dissociated by rigorous vortexing for 1 minute using the Precellys 24 Tissue Homogenizer and lysates immediately frozen at -80°C. RNA extraction was carried out using the Qiagen RNeasy kit according to the manufacturer’s protocol.

#### RTqPCR

RNA was isolated using the Qiagen RNeasy kit and cDNA was generated by using the QuantiTect reverse transcription kit (Qiagen) according to the manufacturer’s instructions. RTqPCR was performed with human or mouse Taqman Gene Expression Assay probes on an Applied Biosciences QuantStudio6 Flex Real-time PCR machine and relative quantification was performed using QuantStudio Real-time PCR software. Each reaction was performed in triplicate. Relative expression levels were normalized to *B2m/B2M*, *Gapdh/GAPDH,* or *Tmem199/TMEM199* endogenous controls, and ≥2 controls were used in each experiment. In control 3T3 fibroblast RNA samples, the *Mmp3* and *Il1a* probes did not amplify during the run. Consequently, RQ values were calculated using an assumed CT value of 40 for the control samples, based on the maximum number of cycles used. Confidence intervals were set at 95% for all assays.

#### NanoString gene expression analysis

RNA was extracted from frozen lung tissue as described above. 80 ng RNA was hybridized with the Mouse PanCancer Immune panel or Mouse PanCancer Pathways panel and processed using the nCounter SPRINT Profiler (NanoString) following the manufacturer’s instructions. Raw NanoString data was pre-processed using R package NanoStringNorm (v1.2.1)^74^ and further normalized using voom (TMM normalization) followed by differential gene expression analysis with R package limma (v3.40.6).^75^ Genes with absolute log_2_ fold change ≥ 0.585 and FDR adjusted *p* value < 0.05 were considered significantly different. Principle component analysis (PCA) was performed using R package FactoMineR (v2.3). All analyses were performed in R statistical programming language (v3.6.14). Visualizations were generated using in-house plotting libraries. Immune cell population abundance was performed as described previously.^76^ In brief, NanoString curated genesets representing specific cell types were used. For each cell type, genesets with more than two genes were further reduced to the largest positively correlated cluster of genes by running hierarchical clustering on Spearman’s correlation distance, followed by identification of optimal number of clusters using Silhouette score. Genes were kept if they all showed pairwise Spearman’s *P* > 0.5.

#### Induction of fibroblast senescent *in vitro*

Fibroblasts were cultured in DMEM with 10% FBS in 75 cm^2^ flasks to ∼75% confluence, then medium was replaced with DMEM with 10% FBS containing chemotherapeutic agents. Fibroblasts were incubated in medium containing the drug for 24 hours, washed twice with PBS and then cultured in fresh DMEM with 10% FBS. Senescent fibroblasts were maintained in the same flasks for up to 6 months without further passage, growth medium was replaced weekly with fresh DMEM plus 10% FBS. To monitor senescence, fibroblasts induced into senescence as described above were seeded in 6-well or 96-well plates at 80-90% confluency 7-21 days following drug withdrawal and incubated for 24 hours before staining. Control fibroblasts were seeded into a 6-well plate and cultured for 2-3 days until they matched the confluency of the senescent fibroblasts. Fibroblasts were washed with PBS, fixed for 10 minutes with 2% paraformaldehyde and 0.2% glutaraldehyde, washed twice with PBS and 1.5 mL staining solution (SA-β-Gal histochemical staining kit, Sigma CS0030) containing X-Gal was added. Plates were sealed with Parafilm and incubated at 37°C in a non-humidified incubator at atmospheric CO_2_ concentration. After 24 hours the cells were washed with PBS and imaged at 100x magnification. For live cell imaging of GFP signal, cells were imaged using either the EVOS fluorescence microscope or the Incucyte live cell imaging fluorescence microscope using Excitation 470/Emission 525 wavelengths.

#### Conditioned media (CM)

CM was collected from chemotherapy-treated fibroblasts 7-30 days following chemotherapy withdrawal. Chemotherapy-treated or control fibroblasts were washed twice and then cultured in serum-free DMEM. After 24 hours, CM was collected, centrifuged at 300*g,* filtered through a 0.2 μm pore filter and used fresh or stored at -80°C for future use. CM was used within 6 months of freezing and was filtered post-thawing.

#### Cytokine arrays

CM was freshly collected as described above from chemotherapy-treated human and mouse fibroblasts or from control fibroblasts. CM was collected independently from two flasks per condition, and 100 μL added to duplicate RayBiotech G2000 array slides. Wash and incubation steps were performed according to the manufacturer’s protocol. Fluorescence signal detection at 532 nm was performed on a GenePix 4000B array scanner. Median fluorescence intensity (MFI) was quantified using ImageJ software. Fold changes were calculated by dividing the mean MFI for the two chemotherapy-treated CM replicates by the mean MFI for the two control replicates for each fibroblast type.

#### Phosphoprotein arrays

Tumor cells were seeded into ultra-low attachment U-bottom 96-well plates in DMEM with 2% FBS at density of 12,500 cells per well and incubated for 48 hours. 192 spheroids were divided into 2 groups of 96 and placed into 15 mL Falcon tubes, washed once with PBS and then incubated in 12 mL serum-free DMEM for 2 hours at 37°C on a roller. Spheroids were centrifuged at 260*g*, medium removed and replaced with 10 mL of control or chemotherapy-treated fibroblast CM and incubated for 30 minutes at 37°C on a roller. Spheroids were then washed 2X in 10 mL ice-cold PBS then lysed in 100 μL Cell Signalling Technologies lysis buffer containing 1 mM PMSF. Cell lysates were sonicated in an ice-cold water bath for 10 minutes, frozen to -80°C for 30 minutes, thawed, centrifuged at 16,000*g* for 10 minutes at 4°C, the supernatant was transferred to a fresh tube on ice and the pellet discarded. Protein concentration was measured using the Millipore Direct Detect Spectrometer according to the manufacturer’s protocol. Protein samples were diluted in array blocking buffer and 150 μL was added to the Pathscan antibody-array slides (Cell Signalling Technologies #7982). Wash and incubation steps, and sample detection using a chemi-luminescent HRP substrate were performed according to the manufacturer’s protocol. Luminescence was imaged using the Odyssey Fc Imaging System. Median fluorescence intensity (MFI) was quantified using ImageJ software. Fold changes were calculated by dividing the mean MFI for the two technical replicate spots for each target in the senescent CM treated spheroids by the mean MFI for the two technical replicate spots for each target in the control CM treated spheroids.

#### Co-culture spheroid assays

1,000 tumor cells and 1,000 fibroblasts were co-seeded into ultra-low attachment U-bottom 96-well plates and incubated for 24 hours. Spheroids were treated with doxorubicin (0 – 0.85 μM) and incubated for a further 72 hours before imaging on an EVOS microscope. Alternatively, 5,000 tumor cells and 5,000 fibroblasts were co-seeded, incubated for 24 hours before addition of 0.17 μM doxorubicin or vehicle. 72 hours later, spheroids were treated with 4.5 μM navitoclax or vehicle and incubated for a further 72 hours before being imaged and assayed by CellTiter-Glo.

#### BCL-2 family inhibitors dose response assays

Chemotherapy-treated fibroblasts were seeded into 96-well or 24-well plates for CellTiter-Glo or crystal violet analysis, respectively. Chemotherapy-treated fibroblasts were seeded at ∼95% confluence in parallel with control fibroblasts at ∼70% confluence and incubated for 24 hours prior to treatment with BCL-2-family inhibitors, alone or in combination, as indicated for 72 hours before assaying cell viability by CellTiter-Glo or staining with 0.5% crystal violet in methanol.

### Quantification and statistical analysis

Statistics were performed using GraphPad Prism 8 or 9. All data is represented as the mean value ± standard error of the mean. Comparisons between two groups were made using two-tailed, unpaired Student's *t*-tests. If there was a significant difference between the standard deviations of the two groups, then Welch’s correction was applied. If the analysis did not pass the Shapiro-Wilk normality test, then groups were analyzed by Mann-Whitney *U*-test. If more than 2 groups were compared One-way ANOVA analysis was performed with Dunnett’s correction for multiple comparisons. If a significant difference between the standard deviations of the means was measured then the Brown-Forsythe and Welch correction was applied. If data did not pass the Shapiro-Wilk normality test then a Kruskal-Wallis ANOVA was performed with Dunn’s correction for multiple comparisons.
