# Supplementary material for: Therapy-induced normal tissue damage promotes breast cancer metastasis
Source: iScience. 2023 Nov 22;27(1):108503. doi: 10.1016/j.isci.2023.108503 (PMC10755366; doi:10.1016/j.isci.2023.108503)
Supplement: Document S1. Figures S1–S5 and Tables S1–S4 [file mmc1.pdf]

iScience, Volume *27*

Supplemental information

Therapy-induced normal tissue

damage promotes breast cancer metastasis

Douglas W. Perkins, Ivana Steiner, Syed Haider, David Robertson, Richard Buus, Lynda O'Leary, and Clare M. Isacke

SUPPLEMENTARY MATERIAL

Figures S1 - S5

Figure S1 | Chemotherapy limits tumor growth *in vivo*, related to Figure 1

Figure S2 | Effects of chemotherapy treatment on immune cell populations in the lung, related to Figure 2

Figure S3 | Response of chemotherapy-treated fibroblasts to BCL-2 family inhibitors *in vitro*, related to Figure 5

Figure S4 | Principal component analysis (PCA) of NanoString PanCancer Immune and PanCancer Pathways panel data, related to Figures 2 and 7

Figure S5. | Effects of chemotherapy and navitoclax treatment on immune cell populations in the lung and higher power images from Figure 7.

Tables S1 - S4

Table S1 | NanoString (PanCancer Immune panel) differentially expressed genes, related to Figures 2 and 7.

Table S2 | NanoString (PanCancer Pathways panel) differentially expressed genes, related to Figures 2 and 7.

Table S3 | RTqPCR probes, related to STAR methods reagents

Table S4 | Antibodies, related to STAR methods reagents

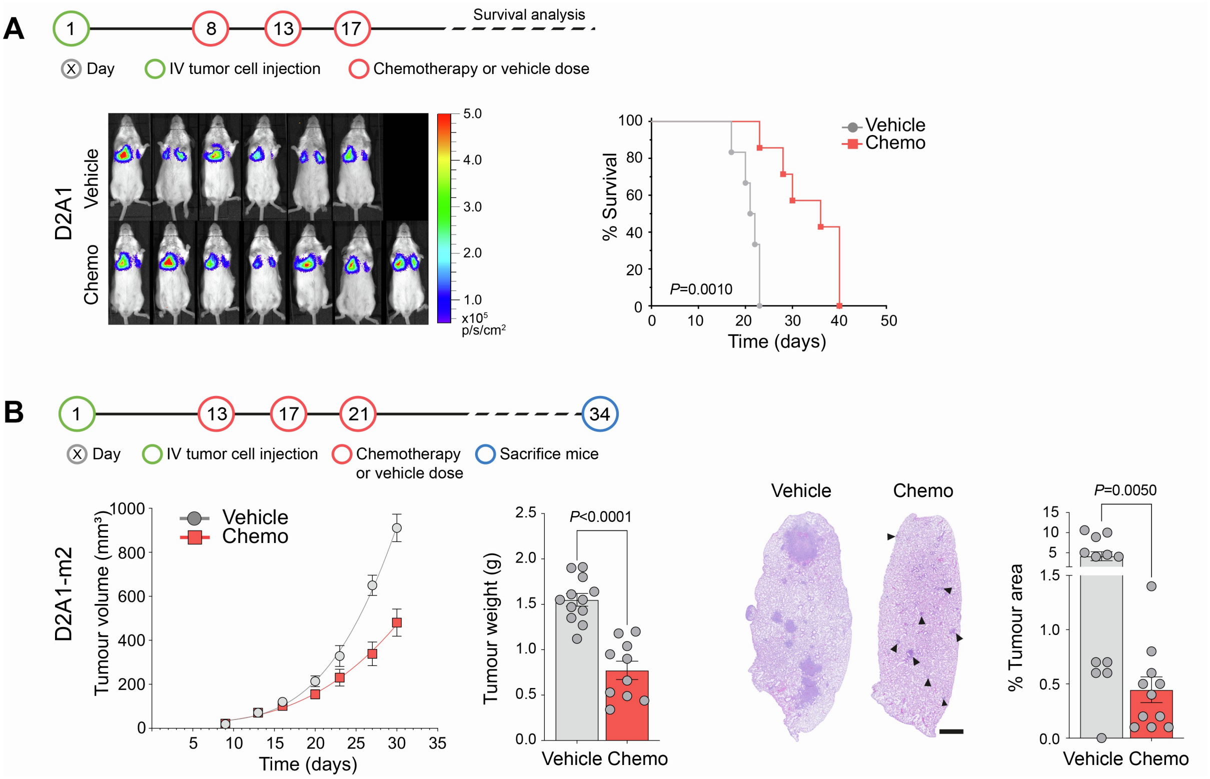

Figure S1. Chemotherapy limits tumor growth *in vivo,* related to Figure 1. **A** Experimental timeline of BALB/c mice inoculated intravenously on Day 1 with 5 x 10^5 D2A1-mChLuc2 tumor cells (n=6-7 mice per group) followed by combination chemotherapy or vehicle treatments starting on Day 8. Shown are IVIS images taken ~90 minutes after tumor cell injection and Kaplan-Meier survival analysis (Log-rank (Mantel-Cox) test). Mice were culled individually when thoracic IVIS signal exceeded 1 x 10^9 photons per second or if a mouse showed signs of ill health. **B** Experimental timeline of BALB/c mice inoculated orthotopically (4th mammary fat pad) on Day 1 with 2 x 10^5 D2A1-m2 tumor cells (n=10-12 mice per group). Shown are: primary tumor growth measured twice weekly; tumor weight at necropsy on Day 34 (±SEM, unpaired t-test); representative images of lung H&E stained sections, arrowheads indicate micrometastatic deposits in chemotherapy-treated lungs (scale bar, 1 mm); quantification of spontaneous metastasis to the lungs in 3 lung sections per mouse (mean % tumor burden per lung section ±SEM, unpaired t-test).

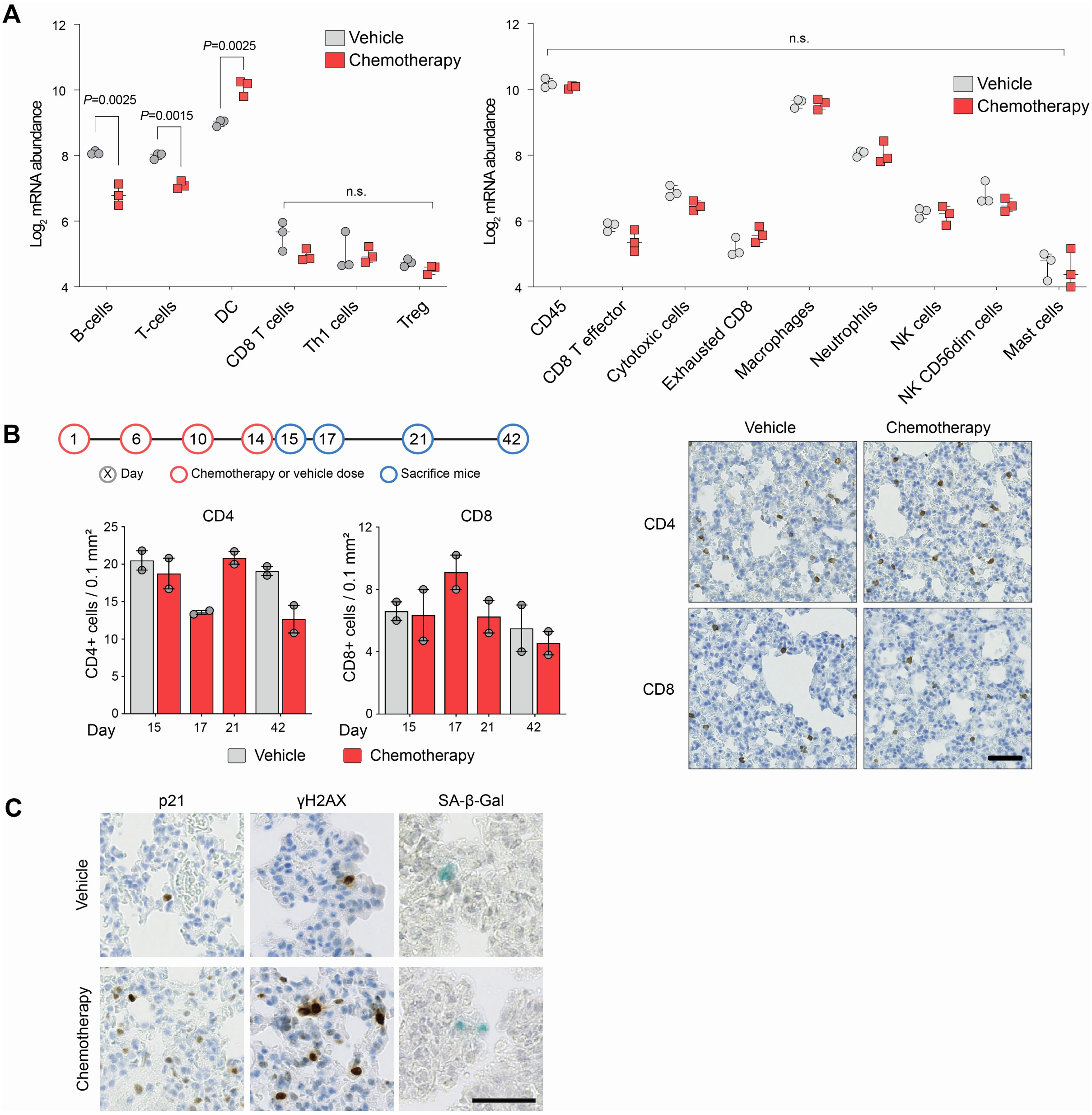

Figure S2. Effects of chemotherapy treatment on immune cell populations in the lung, related to Figure 2. **A** NanoString immune cell population abundance signatures for data shown in Figure 2A,B (median values, ±min to max; multiple unpaired *t*-tests, n.s. = non-significant). **B** BALB/c mice treated with a course of chemotherapy or vehicle as indicated and sacrificed on Day 15, 17, 21 and 42 (1, 3, 7 and 28 days after the last treatment dose). Lung sections were stained for CD4 or CD8, and the number of positively stained cells was quantified in a blinded fashion, using ImageJ software. Positive cells were counted in 6, randomly selected, 0.1 mm^2 fields of view per lung section. Shown are the mean number of $CD4^+$ and $CD8^+$ cells per 0.1 mm^2 ±SEM. Representative images are shown from vehicle-treated mice sacrificed on Day 15 and from chemotherapy-treated mice sacrificed on Day 21. Scale bar, 50 μm. **C** Higher power images from Figure 2D (scale bar, 50 μm).

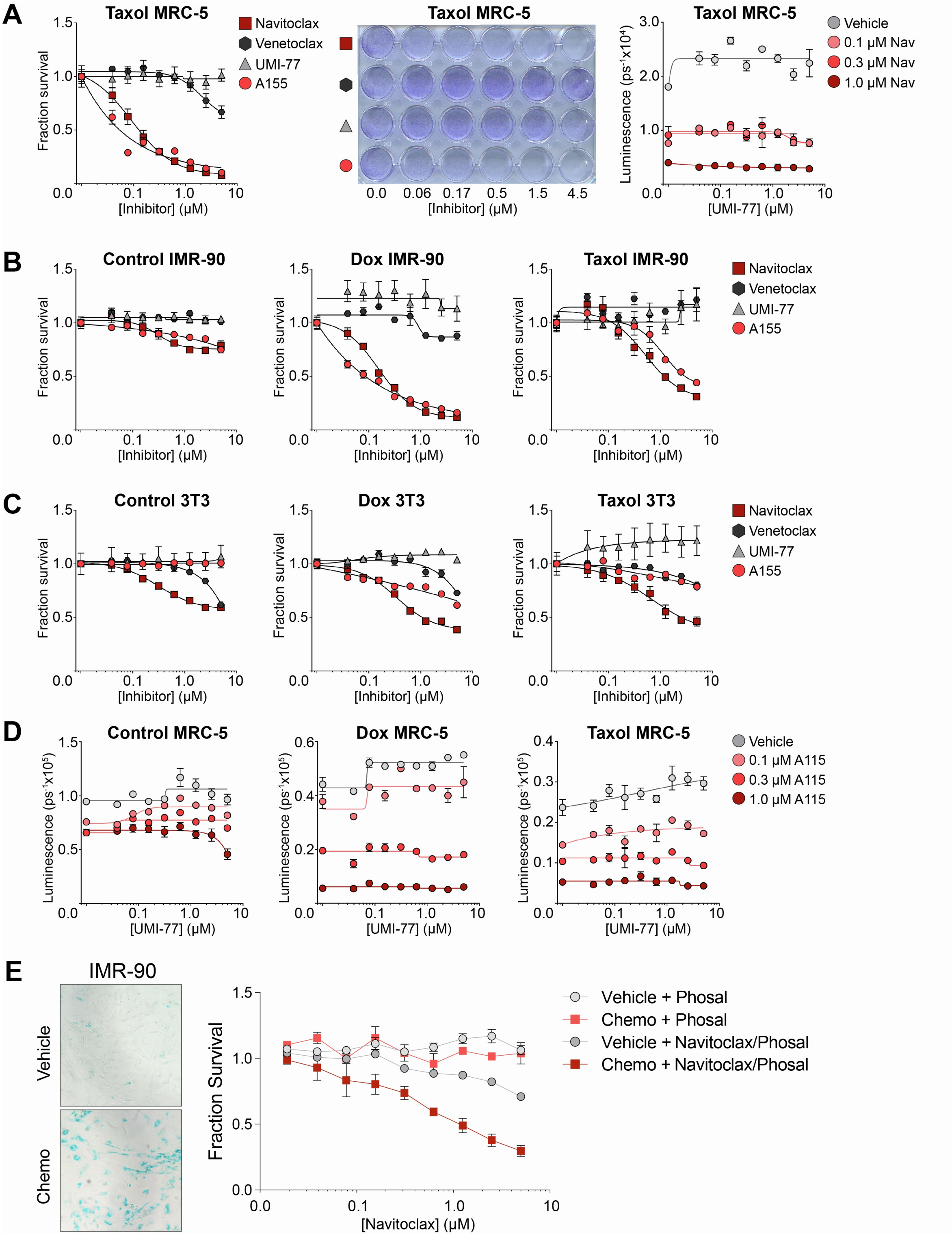

Figure S3. Response of chemotherapy-treated fibroblasts to BCL-2 family inhibitors *in vitro*, related to Figure 5. **A** MRC-5-146a fibroblasts were induced to senescence by 24 hour treatment with 1.2 μm docetaxel. 9 days after treatment withdrawal, chemotherapy-treated or control fibroblasts were plated into 96-well plates (n=3 wells per condition) or 24 well plates (n=1 well per condition). After 24 hours fibroblasts were treated with individual BCL-2 family

inhibitors, or with a range of concentrations of UMI-77 in combination with 3 concentrations of navitoclax or DMSO control. 72 hours later, cell viability was measured by CellTiter-Glo (left and right panels, mean values ±SEM) or plates were stained with crystal violet (middle panel). **B** IMR-90-146a fibroblasts were treated for 24 hours with 1.7 μm doxorubicin or 1.2 μm docetaxel. 20 days after treatment withdrawal, chemotherapy-treated or control fibroblasts were plated into 96-well plates (n=3 wells per condition). After 24 hours fibroblasts were treated with BCL-2 family inhibitors as indicated and incubated for 72 hours. Cell viability was measured by CellTiter-Glo (mean values ±SEM). **C** 3T3-146a fibroblasts were treated for 24 hours with 0.17 μm doxorubicin or 0.12 μm docetaxel. 7 days after treatment withdrawal, chemotherapy-treated or control fibroblasts were plated into 96-well plates (n=3 wells per condition). After 24 hours fibroblasts were treated with BCL-2 family inhibitors as indicated and incubated for 72 hours. Cell viability was measured by CellTiter-Glo (mean values ±SEM). **D** MRC-5-146a fibroblasts were treated for 24 hours treatment with 1.7 μm doxorubicin or 1.2 μm docetaxel. 10 days after treatment withdrawal, chemotherapy-treated or control fibroblasts were plated in 96-well plates (n=3 wells per condition). After 24 hours fibroblasts were treated with range of concentrations of UMI-77 alone or in combination with 3 concentrations of A115 and incubated for 72 hours. Cell viability was measured by CellTiter-Glo (mean values ±SEM). **A-D** Equivalent results were obtained in 2 or 3 independent experiments. **E** IMR-90 fibroblasts were treated for 24 hours with 0.17 μm doxorubicin. 17 days after treatment withdrawal senescent cells were stained and visualized using SA-β-Gal staining kit. 7 days after treatment withdrawal, chemotherapy-treated or control fibroblasts were plated into 96-well plates (n=2 wells per condition). After 24 hours fibroblasts were treated with Navitoclax reconstituted in Phosal and incubated for 72 hours. Cell viability was measured by CellTiter-Glo (mean values ±SEM).

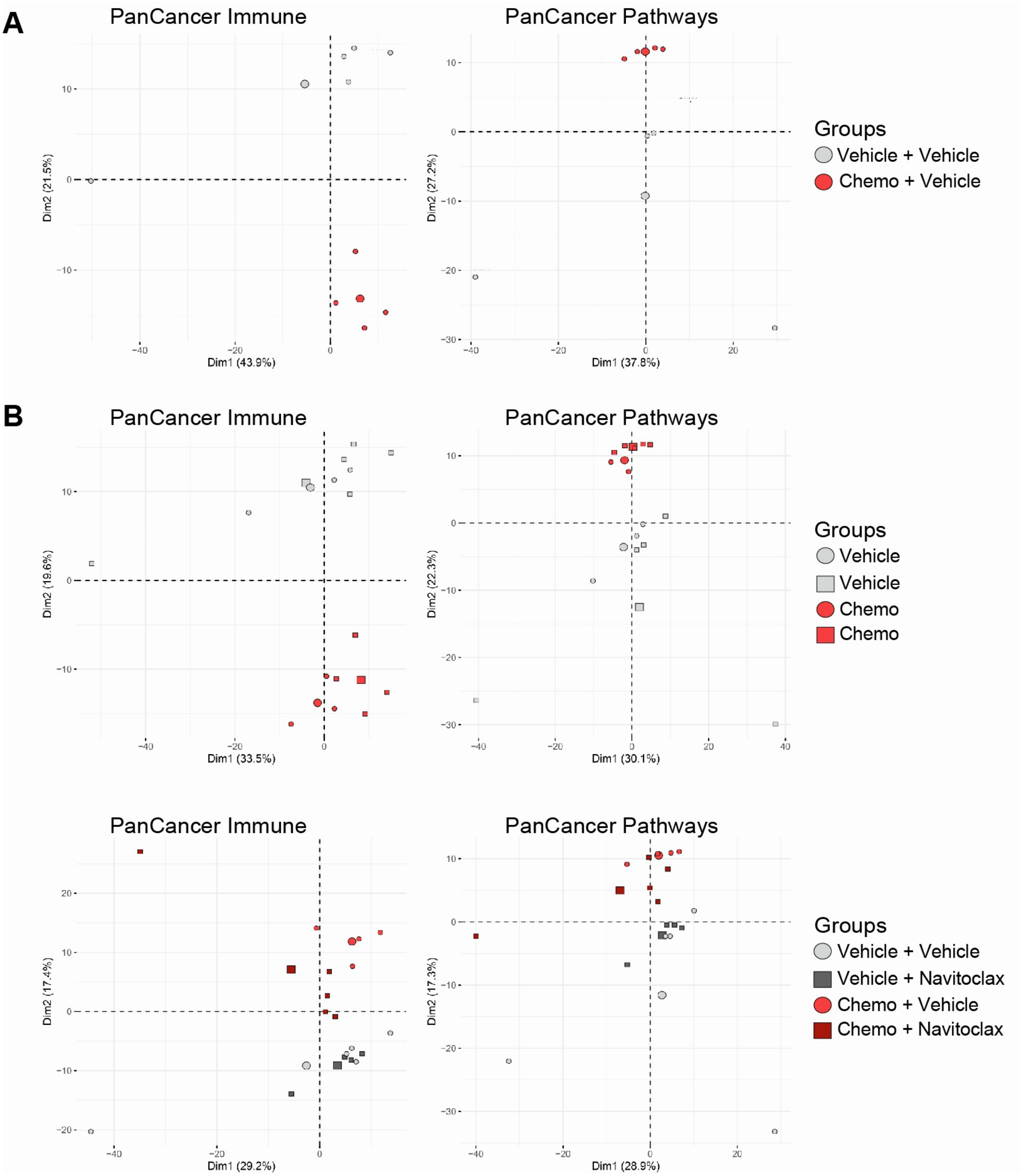

Figure S4. Principal component analysis (PCA) of NanoString PanCancer Immune and PanCancer Pathways panel data, related to Figures 2A,B and 7A-C. Shown are the PCA plots based on expression of the 750 target genes in each panel from: **A** Vehicle + Vehicle and Chemo + Vehicle mice from Figure 7B and C; **B** Panel A samples combined with Vehicle and Chemo samples from Figure 2B; **C** All samples from Figure 7B-C.

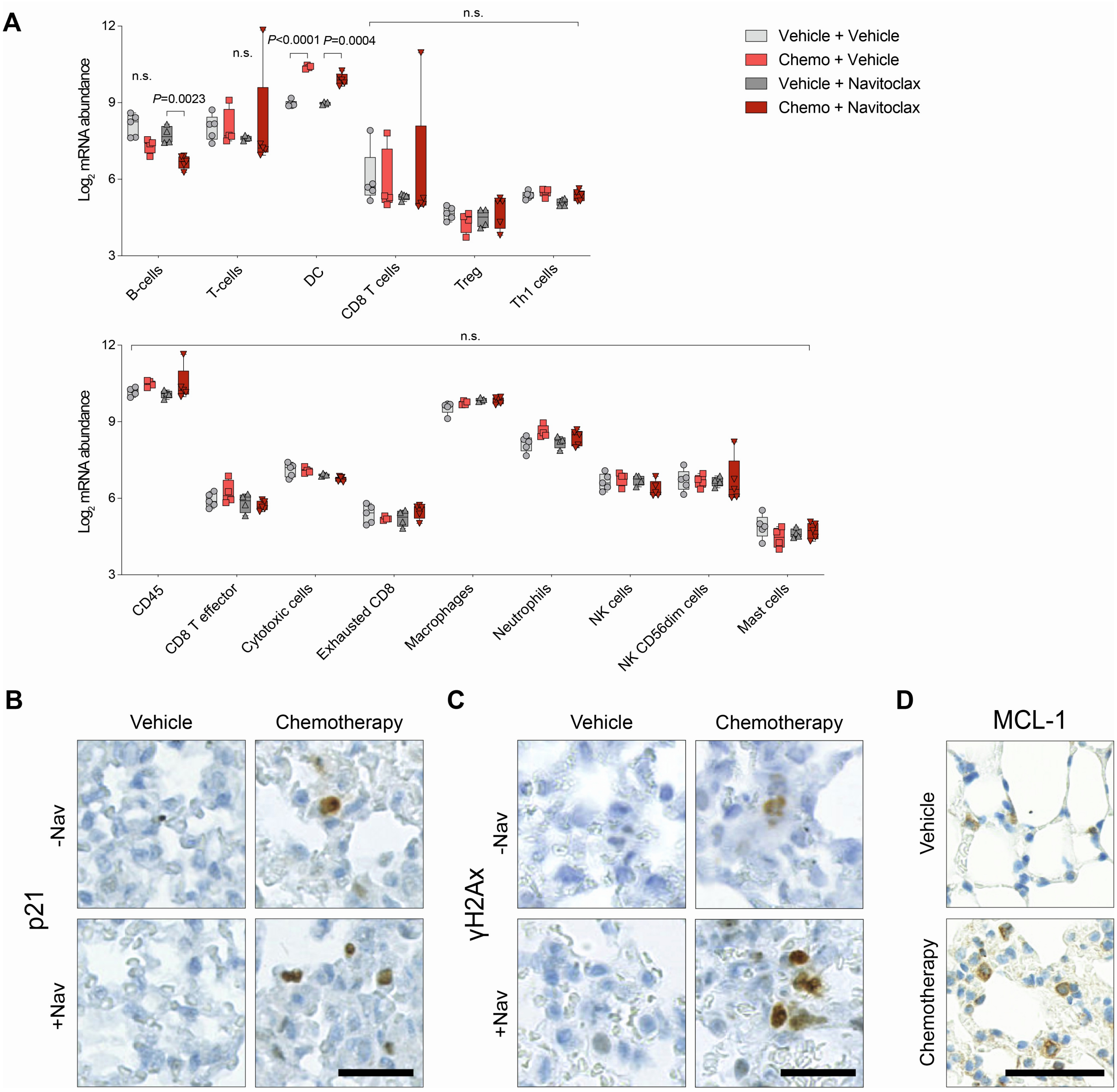

Figure S5. Effects of chemotherapy and navitoclax treatment on immune cell populations in the lung and higher power images from Figure 7. **A** NanoString immune cell population abundance signatures related to Figure 7B-C. (median values, ±min to max; multiple unpaired *t*-tests, n.s. = non-significant). **B - D** Higher power images from Figure 7D, Figure 7E and Figure 7G, respectively (scale bar, 50 μm).

Table S1: Top 150 & bottom 50 NanoString PanCancer Immune panel hits ranked by fold change (FC). Related to Figures 2&7

Fig. 2B Vehicle vs Chemo			Fig.7B Vehicle vs Chemo			Fig. 2B+7B Veh. vs Chemo			Fig. 7C Vehicle ± Nav			Fig. 7C Chemo ± Nav		
Factor	FC	adj.P.Val	Factor	FC	adj.P.Val	Factor	FC	adj.P.Val	Factor	FC	adj.P.Val	Factor	FC	adj.P.Val
Cdkn1a	4.87	1.38E-04	Ccl2	6.09	3.62E-07	Ccl2	5.31	3.27E-08	Mx1	3.48	6.28E-01	Mpped1	6.63	6.55E-02
Ccl8	4.70	6.72E-03	Cxcl10	4.79	5.02E-05	Cxcl10	4.46	2.69E-06	Itga2b	2.75	1.12E-01	Cfd	3.92	7.82E-01
Ccl2	4.63	1.38E-04	Cdkn1a	3.59	6.69E-05	Cdkn1a	4.18	4.14E-07	Mpped1	1.88	9.10E-01	Itga2b	2.52	8.69E-02
Cxcl10	4.16	3.06E-03	C3ar1	3.52	5.21E-07	Ccl8	3.66	3.15E-04	Ppbp	1.84	8.78E-01	Rag1	2.27	8.52E-01
Ccl7	3.13	1.17E-03	Ccr3	3.36	4.35E-01	Ccl7	3.24	1.25E-06	Defb1	1.77	9.90E-01	Slamf1	2.17	1.12E-01
C8a	3.02	3.99E-01	Ccl7	3.36	4.88E-05	Ccr3	2.77	4.21E-01	Crp	1.68	8.78E-01	A2m	2.03	3.26E-01
Ccl12	3.00	3.12E-03	Lif	2.97	1.08E-08	Ccl12	2.75	1.62E-05	Il13ra2	1.66	8.78E-01	Bcl2l1	1.81	6.94E-04
Il19	2.69	3.35E-02	Ccl8	2.85	2.52E-02	C3ar1	2.74	2.64E-07	Ctsg	1.63	8.78E-01	Marco	1.78	2.04E-01
Birc5	2.69	2.89E-01	Usp9y	2.75	4.35E-01	Lif	2.52	3.36E-09	Tnfsf4	1.62	8.78E-01	Dmbt1	1.77	7.25E-01
Mbl2	2.59	3.90E-01	Cx3cr1	2.74	5.21E-07	Trem2	2.43	9.29E-08	Serpinb2	1.60	9.73E-01	Il2ra	1.75	6.03E-01
S100b	2.54	3.33E-01	Il6	2.65	1.36E-02	Usp9y	2.43	3.03E-01	Chit1	1.55	9.90E-01	Ctsg	1.72	3.42E-01
Trem2	2.36	1.38E-04	Ccl17	2.59	5.10E-04	Birc5	2.31	7.74E-02	Slamf1	1.55	8.78E-01	H2-DMb1	1.65	4.94E-01
Ccr3	2.29	8.25E-01	Ccl12	2.53	6.23E-04	Ccl17	2.30	1.02E-04	Il1rapl2	1.54	8.96E-01	Rorc	1.60	7.70E-01
Prg2	2.21	1.44E-01	Trem2	2.49	2.15E-06	Cx3cr1	2.25	3.39E-07	Il4	1.52	8.78E-01	Tnfrsf8	1.58	6.30E-01
Egr1	2.17	4.31E-03	C1qa	2.22	1.91E-04	Il6	2.10	1.25E-02	Il11	1.48	8.78E-01	Masp2	1.57	8.05E-02
Rag1	2.15	8.92E-01	S100a8	2.21	4.50E-03	C1qb	2.09	2.52E-05	Lyve1	1.47	8.78E-01	Defb1	1.55	9.07E-01
Thbs1	2.15	1.67E-02	Ccl9	2.19	4.88E-05	C1qa	2.09	1.54E-05	Klrb1c	1.45	8.78E-01	Cd8a	1.54	8.87E-01
Lif	2.14	1.28E-04	C1qb	2.17	4.03E-04	C4b	1.96	1.10E-06	Cd70	1.44	9.10E-01	Klra3	1.54	4.26E-01
Usp9y	2.14	7.11E-01	Ccr5	2.11	4.73E-04	Cd80	1.86	1.28E-03	Raet1	1.42	8.78E-01	Ppbp	1.51	7.06E-01
C3ar1	2.13	3.69E-03	Clec5a	2.07	5.35E-04	Ccl9	1.85	2.29E-05	Trem2	1.41	5.74E-01	Crp	1.51	5.17E-01
Cd80	2.10	1.95E-02	Ccr2	2.04	6.99E-07	Cfb	1.83	3.53E-03	C3ar1	1.40	8.78E-01	Lyve1	1.51	3.35E-01
Ccl17	2.05	3.51E-02	Birc5	1.98	3.25E-01	Clec5a	1.83	1.89E-04	C8a	1.39	9.90E-01	H60a	1.50	6.03E-01
Cfb	2.03	5.18E-02	Cxcr1	1.92	8.79E-02	Col4a1	1.79	2.69E-06	Klra21	1.39	8.96E-01	Mertk	1.50	6.96E-02
Cxcl9	2.02	1.00E-01	C4b	1.91	6.90E-05	C8a	1.79	4.02E-01	Il22ra2	1.38	8.78E-01	Cdk1	1.48	3.38E-01
C4b	2.01	5.22E-04	Il1rapl2	1.86	2.31E-01	Il19	1.77	2.49E-02	Epsti1	1.38	9.10E-01	C9	1.48	7.82E-01
C1qb	2.00	8.64E-03	Mx1	1.85	4.26E-01	Ccr5	1.76	5.38E-04	Tpsab1	1.37	9.10E-01	Tcf7	1.47	8.42E-01
C1qa	1.96	7.78E-03	Ccl25	1.78	6.77E-01	Mbl2	1.74	2.89E-01	Ambp	1.36	8.78E-01	Twist1	1.45	8.87E-01
Mefv	1.93	1.63E-01	Col4a1	1.72	2.31E-04	Thbs1	1.72	3.31E-03	Ada	1.33	9.90E-01	Birc5	1.45	7.90E-01
Rsad2	1.92	5.03E-02	Ptgdr2	1.69	1.12E-02	C3	1.71	3.16E-03	Il22	1.32	8.78E-01	Tigit	1.44	4.97E-01
C3	1.90	4.01E-02	Cxcl1	1.69	3.68E-02	Mefv	1.71	3.13E-02	Bst1	1.31	8.78E-01	Klrb1	1.44	7.13E-01
Dmbt1	1.86	6.10E-01	Zbp1	1.69	1.36E-02	Ccr2	1.70	1.10E-06	Cdk1	1.31	8.78E-01	Gfi1	1.44	7.82E-01
Il24	1.86	3.00E-01	Cxcl5	1.69	5.43E-02	S100a8	1.69	9.79E-03	C2	1.31	8.78E-01	Tpsab1	1.42	7.19E-01
Igll1	1.86	1.43E-01	Aicda	1.67	3.40E-01	Cxcl5	1.69	1.33E-02	Havcr2	1.30	8.96E-01	Il10	1.41	4.94E-01
Col4a1	1.85	9.25E-04	Il2rb	1.66	3.32E-03	Cxcr1	1.67	8.48E-02	Itgb3	1.29	8.78E-01	Il17rb	1.41	8.24E-01
Cx3cr1	1.85	4.34E-03	Cfb	1.66	6.15E-02	Prg2	1.67	8.03E-02	Pmch	1.29	9.90E-01	Cd5	1.40	8.65E-01
Cxcl11	1.83	4.51E-01	Cd80	1.65	4.69E-02	S100b	1.66	3.12E-01	Cxcl14	1.29	8.78E-01	Il23r	1.37	4.97E-01
Il11	1.81	3.17E-01	Ifit3	1.65	1.56E-03	Ulbp1	1.64	3.13E-02	Chil3	1.29	9.18E-01	Klra5	1.37	7.16E-01
Glycam1	1.78	7.17E-01	Ulbp1	1.65	1.12E-01	Cxcl1	1.63	9.79E-03	Rsad2	1.29	8.78E-01	C7	1.36	4.97E-01
Klra3	1.77	1.90E-01	Itgam	1.64	7.24E-03	Cxcl11	1.63	2.13E-01	Ccl22	1.28	9.71E-01	Il12rb1	1.36	7.82E-01
Camp	1.77	2.71E-01	Ccl19	1.64	4.07E-02	Zbp1	1.63	3.53E-03	Tdo2	1.28	8.78E-01	Igll1	1.36	6.11E-01
Tnfrsf10b	1.77	4.99E-03	Rrad	1.60	1.61E-02	Pdcd1	1.61	1.62E-01	Smpd3	1.28	9.90E-01	Ada	1.36	9.95E-01
Mpo	1.77	5.59E-01	Fcer1g	1.59	3.66E-04	Ifit3	1.60	1.41E-04	Vwf	1.27	6.83E-01	Notch1	1.35	1.43E-02
Mpped1	1.74	6.41E-01	Klra1	1.59	2.62E-01	Ccl19	1.59	1.02E-02	Fcgr4	1.26	8.78E-01	Ccl25	1.35	9.49E-01
Ptgs2	1.73	6.47E-02	Tnfrsf1b	1.59	4.57E-04	Cxcl9	1.59	4.34E-02	Clec4n	1.26	8.78E-01	Lck	1.35	8.87E-01
Il12rb1	1.71	5.36E-01	Siglec1	1.58	8.57E-03	Camp	1.57	6.84E-02	Gzmb	1.26	8.78E-01	Cd70	1.35	7.82E-01
Pdcd1	1.70	4.59E-01	Chil3	1.57	1.84E-01	Rsad2	1.56	1.88E-02	Ifit3	1.26	8.78E-01	Atg16l1	1.34	5.83E-02
Cxcl5	1.69	1.96E-01	Ly86	1.57	6.82E-03	Socs3	1.55	4.55E-03	Lyz2	1.26	8.78E-01	Fas	1.34	8.05E-02
Bax	1.69	1.38E-04	Socs3	1.56	2.38E-02	Il11	1.52	1.69E-01	Cxcr4	1.26	8.78E-01	Ctsl	1.34	5.03E-01
Klra4	1.68	3.66E-01	Plau	1.56	5.32E-04	Raet1	1.52	8.35E-03	Cd84	1.26	8.78E-01	Flt3l	1.33	1.75E-01
Il6	1.67	4.10E-01	Sh2d1a	1.56	6.75E-01	Bax	1.52	3.06E-06	Msln	1.26	8.78E-01	Cd247	1.33	8.73E-01
Ccr8	1.64	3.34E-01	Tdo2	1.55	8.24E-02	Siglec1	1.51	2.59E-03	Prdm1	1.25	8.78E-01	Foxp3	1.33	6.25E-01
Ifnl2	1.64	2.47E-01	Ccr1	1.55	2.10E-02	Cd14	1.49	1.22E-04	Lrrn3	1.25	8.78E-01	Il17b	1.33	6.11E-01
Ulbp1	1.63	2.89E-01	C3	1.54	7.08E-02	Tnfrsf17	1.49	2.71E-01	Fcer1g	1.25	8.75E-01	Fcer1a	1.33	6.57E-01
Lag3	1.62	1.92E-01	Csf3r	1.53	4.88E-02	Fcer1g	1.48	1.08E-04	Abcg1	1.25	8.78E-01	Ccl26	1.33	6.20E-01
Clec5a	1.61	7.90E-02	Slc11a1	1.53	1.22E-02	Mx1	1.47	4.63E-01	Tnfsf14	1.24	8.78E-01	Cd3e	1.32	8.95E-01
Raet1	1.61	1.01E-01	Ifi44l	1.52	5.89E-02	Pdgfrb	1.47	2.28E-05	Cxcl10	1.24	9.90E-01	Il11	1.32	7.57E-01
Cxcl1	1.58	2.04E-01	Cd7	1.51	3.83E-02	Isg15	1.47	4.64E-03	Gpr44	1.24	8.96E-01	Bcl6	1.32	3.42E-01
Tmem173	1.56	8.64E-03	Pdcd1	1.51	3.80E-01	Jak3	1.47	9.96E-05	Csf2	1.24	8.78E-01	Cd163	1.32	6.11E-01
Nt5e	1.56	3.96E-02	Mefv	1.51	2.40E-01	Plau	1.46	1.37E-04	Ifna2	1.23	9.90E-01	Ctla4	1.30	6.12E-01
Ccl9	1.56	4.38E-02	Il4	1.50	2.87E-01	Igll1	1.46	1.04E-01	Pdcd1	1.23	9.90E-01	Lrrn3	1.30	3.26E-01
Zbp1	1.56	1.48E-01	Fn1	1.50	1.31E-02	Ptgdr2	1.45	1.58E-02	Fcgr1	1.23	8.78E-01	Il1rapl2	1.30	8.26E-01
Fos	1.56	7.06E-01	Isg15	1.49	2.10E-02	Ccl25	1.45	7.29E-01	Hsd11b1	1.22	8.78E-01	Nfkbia	1.29	3.42E-01
Ifit3	1.55	2.80E-02	C2	1.49	1.24E-01	Itgam	1.45	8.59E-03	Klra2	1.22	8.78E-01	Cd244	1.28	6.20E-01
Ccl19	1.55	1.96E-01	Il13ra2	1.49	4.99E-01	Tnfrsf10b	1.45	1.82E-03	Xcl1	1.22	9.18E-01	Nfatc3	1.28	5.96E-01
Socs3	1.54	1.37E-01	Klra2	1.48	4.92E-02	Il24	1.44	2.45E-01	Il12b	1.22	9.46E-01	Ccl28	1.28	7.69E-01
Jak3	1.53	6.03E-03	Tnfrsf17	1.48	4.52E-01	Ifi44l	1.43	4.04E-02	Card9	1.22	8.96E-01	Klra15	1.28	8.13E-01
Col3a1	1.52	2.82E-01	Gzmb	1.48	9.85E-02	Tnfrsf1b	1.43	3.19E-04	Cd68	1.22	8.78E-01	Zap70	1.28	8.42E-01
Tnfsf15	1.51	1.37E-01	Gzma	1.48	8.24E-02	Rag1	1.43	8.60E-01	Il10	1.21	9.46E-01	Atf1	1.27	1.35E-01
H2-Q2	1.50	2.39E-01	Cd14	1.47	2.39E-03	Ccr1	1.42	1.44E-02	F13a1	1.21	9.68E-01	Pla2g6	1.27	1.75E-01
Cd14	1.50	1.42E-02	Gbp5	1.47	4.07E-02	Ptgs2	1.42	3.72E-02	Ccl6	1.21	9.10E-01	Inpp5d	1.27	8.66E-02
C9	1.50	6.81E-01	Tnfsf14	1.47	2.37E-02	Cd99	1.42	2.22E-03	C4b	1.21	8.78E-01	Mx1	1.27	8.95E-01
Cxcl2	1.50	3.43E-01	Pdgfrb	1.46	5.81E-04	Il2rb	1.41	9.65E-03	Il1rn	1.21	8.78E-01	Smpd3	1.27	9.05E-01
Col1a1	1.50	1.47E-01	Msr1	1.46	1.36E-02	Bst2	1.40	1.37E-04	Fut7	1.20	9.10E-01	Cd96	1.26	8.85E-01
Tnfrsf17	1.49	6.47E-01	Cxcl11	1.46	5.34E-01	Tnf	1.40	1.71E-02	Aicda	1.20	9.90E-01	Cd1d1	1.26	8.52E-01
Foxj1	1.49	3.25E-01	Oasl1	1.46	8.27E-02	Mx2	1.40	6.85E-02	Ccl28	1.20	9.90E-01	Ikzf2	1.26	7.82E-01
Ifna4	1.49	6.32E-01	Hck	1.45	6.83E-03	H2-Q2	1.39	5.94E-02	Nt5e	1.20	8.78E-01	Nup107	1.25	3.42E-01

Nos2	1.48	9.82E-02	Klrb1c	1.45	1.08E-01	Il12rb1	1.39	4.05E-01	Il23r	1.20	9.62E-01	Itgb4	1.25	8.40E-01
Pdgfrb	1.48	4.99E-03	Cd99	1.44	1.29E-02	Csf1r	1.39	2.32E-05	Itgb2	1.19	8.78E-01	Cfh	1.25	4.75E-01
Ccr4	1.47	6.61E-01	Fcgr1	1.43	1.27E-02	Ccr4	1.39	3.93E-01	Lcn2	1.19	9.63E-01	Cd4	1.25	9.36E-01
Tnfrsf8	1.46	7.06E-01	Raet1	1.43	8.13E-02	Nt5e	1.38	9.63E-03	Ceacam1	1.19	8.84E-01	Pdcd1lg2	1.25	7.50E-01
Ccr5	1.46	2.13E-01	Ccl4	1.42	2.87E-01	Ifnl2	1.38	1.49E-01	Cd200r1	1.18	8.78E-01	Klra27	1.25	8.40E-01
Cxcr1	1.46	6.14E-01	Ncf4	1.42	6.83E-03	Il3ra	1.37	5.32E-04	Ctsh	1.18	8.78E-01	Klra6	1.24	7.42E-01
Oas2	1.45	3.42E-01	Il4ra	1.42	1.31E-03	Msr1	1.37	8.59E-03	Cdh1	1.18	8.78E-01	Mme	1.24	7.06E-01
Il3ra	1.45	1.46E-02	Bst2	1.41	1.92E-03	Hcst	1.37	2.23E-01	Ccl8	1.17	9.90E-01	Myc	1.24	8.42E-01
Isg15	1.45	1.44E-01	Il18rap	1.41	6.15E-02	C2	1.37	1.03E-01	Lilra5	1.17	9.18E-01	Elk1	1.24	6.65E-01
Siglec1	1.44	1.46E-01	Il1rl1	1.41	3.93E-02	Il4ra	1.37	1.83E-04	Itgae	1.17	8.96E-01	Ewsr1	1.24	5.83E-02
Clec4a2	1.44	1.27E-01	Jak3	1.40	4.64E-03	Tmem173	1.36	2.22E-03	Hck	1.17	8.78E-01	Ltk	1.24	7.82E-01
Tnf	1.43	1.76E-01	Csf1r	1.40	5.10E-04	Vwf	1.36	1.65E-03	Klra15	1.17	9.90E-01	Thy1	1.24	9.59E-01
Klra6	1.42	4.37E-01	Usp18	1.40	2.52E-02	Il1rl1	1.36	1.37E-02	C7	1.17	9.88E-01	Ifng	1.23	7.90E-01
Ccr2	1.41	2.12E-02	Mx2	1.39	1.77E-01	F13a1	1.36	1.78E-01	Cxcr6	1.16	9.35E-01	Chil3	1.22	8.26E-01
Vegfc	1.41	6.99E-02	Camp	1.39	3.14E-01	Runx3	1.36	5.94E-02	Tnfsf13b	1.16	8.84E-01	Ilf3	1.22	3.26E-01
Cd44	1.41	4.37E-01	F13a1	1.39	2.95E-01	Ly86	1.36	1.37E-02	Bid	1.16	9.90E-01	Il9	1.22	8.34E-01
Il2	1.40	4.43E-01	Vwf	1.39	1.05E-02	Fn1	1.35	1.33E-02	Il13	1.16	9.90E-01	Arg2	1.21	6.03E-01
Mx2	1.40	3.90E-01	Klrg1	1.38	1.84E-01	Fcgr2b	1.35	2.39E-03	Fcgr3	1.16	8.78E-01	Sele	1.21	8.74E-01
Cd99	1.40	1.03E-01	Thbs1	1.38	2.10E-01	Egr1	1.35	4.99E-02	Cd7	1.16	9.46E-01	Egr2	1.21	7.90E-01
Bst2	1.39	1.96E-02	Tnf	1.37	1.00E-01	Col1a1	1.35	4.62E-02	Mrc1	1.15	8.78E-01	Il1r2	1.21	9.49E-01
Csf1r	1.38	6.39E-03	Fcgr2b	1.37	1.36E-02	Slc11a1	1.34	2.23E-02	Ulbp1	1.15	9.90E-01	Cd44	1.21	7.90E-01
Fcer1g	1.38	5.08E-02	Batf	1.37	1.11E-01	Oas2	1.34	1.21E-01	Ctss	1.15	9.46E-01	Il17a	1.21	8.44E-01
Runx3	1.38	3.38E-01	Hcst	1.37	3.83E-01	Tnfrsf8	1.33	5.04E-01	Serping1	1.15	8.78E-01	Spink5	1.20	9.85E-01
Hcst	1.38	6.12E-01	Bax	1.36	2.37E-03	Fas	1.33	4.33E-03	Il18	1.15	9.10E-01	Slamf6	1.20	8.63E-01
Pmch	1.37	7.69E-01	Nlrp3	1.36	5.31E-02	Ifi44	1.33	7.95E-02	Syt17	1.15	9.88E-01	F12	1.20	8.42E-01
Cspg4	1.37	1.72E-01	Csf1	1.36	4.70E-03	Hck	1.32	6.38E-03	Lamp3	1.15	9.81E-01	Il16	1.20	8.03E-01
Plau	1.37	5.18E-02	Ido1	1.35	4.13E-01	Tdo2	1.32	1.43E-01	Tlr8	1.15	8.78E-01	Cxcl13	1.20	9.77E-01
Il17b	1.36	5.68E-01	Trem1	1.35	3.07E-01	Cspg4	1.32	2.29E-02	C1ra	1.15	8.78E-01	Ms4a2	1.20	8.49E-01
Lbp	1.36	1.66E-01	Hamp	1.35	8.24E-01	Klra17	1.32	1.44E-01	Lgals3	1.15	9.10E-01	Csf2	1.20	6.77E-01
Il23r	1.36	5.18E-01	Cd70	1.34	5.96E-01	Tnfrsf11a	1.32	1.59E-03	C6	1.15	9.81E-01	Klrb1c	1.19	7.25E-01
Ifi44l	1.35	4.43E-01	Fas	1.34	2.16E-02	Fcgr1	1.31	1.51E-02	Abcb1a	1.15	8.78E-01	Ccnd3	1.19	8.34E-01
Mnx1	1.35	6.91E-01	Runx3	1.34	2.00E-01	Col3a1	1.31	1.80E-01	Itga2	1.14	9.10E-01	H2-Q10	1.19	8.34E-01
H2-DMb1	1.34	6.96E-01	Klra17	1.33	2.70E-01	Vegfc	1.31	1.12E-02	Cd9	1.14	9.46E-01	Il15ra	1.19	7.42E-01
Fcgr2b	1.34	1.03E-01	Entpd1	1.33	2.18E-03	Aicda	1.30	5.08E-01	Itga6	1.14	8.78E-01	Gzmm	1.18	7.82E-01
Vwf	1.34	9.39E-02	Tnfrsf11b	1.32	1.47E-01	Lcn2	1.30	2.22E-01	Cd38	1.14	9.10E-01	Itk	1.18	9.49E-01
Tnfrsf1a	1.34	6.03E-03	Il1b	1.32	4.13E-01	Il13ra2	1.29	5.69E-01	Usp18	1.14	8.96E-01	Epsti1	1.18	8.87E-01
Ifi44	1.33	3.99E-01	Itgb3	1.32	1.15E-01	Entpd1	1.29	3.12E-04	Ccrl2	1.14	8.78E-01	Plaur	1.18	4.88E-01
F13a1	1.33	6.05E-01	Ifi44	1.32	2.05E-01	Rrad	1.29	9.23E-02	Tnfrsf8	1.14	9.90E-01	Cd59b	1.18	8.10E-01
Nfkb2	1.33	3.48E-02	Tnfrsf11a	1.31	1.38E-02	Gbp5	1.29	8.03E-02	Cfi	1.14	9.90E-01	Ccl6	1.18	7.82E-01
F12	1.33	6.49E-01	Lcn2	1.31	3.71E-01	Il12b	1.28	2.66E-01	Ifit1	1.14	9.46E-01	Ccl11	1.18	9.66E-01
Ltf	1.33	4.92E-01	Csf2rb	1.31	1.19E-02	Sbno2	1.28	3.64E-04	H2-Q2	1.14	9.90E-01	Colec12	1.17	6.61E-01
Ambp	1.33	5.64E-01	Sbno2	1.31	2.53E-03	Csf3r	1.28	1.56E-01	Muc1	1.13	9.35E-01	Mr1	1.17	3.42E-01
Gata3	1.33	4.43E-01	Ccr4	1.31	6.44E-01	Il23r	1.28	2.54E-01	Alcam	1.13	9.10E-01	Cd27	1.17	9.78E-01
Il4ra	1.32	4.00E-02	Klra21	1.30	5.69E-01	Itgb3	1.28	6.51E-02	Lta	1.13	9.90E-01	Dock9	1.17	4.89E-01
Tnfrsf11a	1.32	6.18E-02	Snai1	1.30	3.47E-01	Ncf4	1.27	1.34E-02	Icam1	1.13	9.73E-01	Cx3cl1	1.17	5.68E-01
Il1rl1	1.32	2.76E-01	Il3ra	1.30	2.10E-02	Csf1	1.27	3.15E-03	Il22ra1	1.13	9.90E-01	Yy1	1.16	1.43E-01
Ccr1	1.31	3.85E-01	Emr1	1.30	7.05E-02	Gata3	1.27	1.85E-01	Txnip	1.13	8.96E-01	Creb5	1.16	7.82E-01
Fas	1.31	1.40E-01	H2-Q2	1.29	3.14E-01	Cxcl2	1.27	2.73E-01	Anxa1	1.13	8.78E-01	Tnfsf18	1.16	8.85E-01
Ccl20	1.30	7.11E-01	Cklf	1.28	3.21E-01	Emr1	1.27	3.27E-02	Cx3cl1	1.12	8.96E-01	Cd8b1	1.16	9.92E-01
Klra17	1.30	5.65E-01	Gpr44	1.28	3.44E-01	Tnfrsf1a	1.27	2.25E-04	Il12rb1	1.12	9.90E-01	Lag3	1.16	8.42E-01
S100a8	1.30	6.05E-01	Il11	1.28	6.03E-01	Foxj1	1.26	2.64E-01	Cybb	1.12	9.81E-01	Tlr7	1.16	7.82E-01
Masp1	1.29	4.86E-01	Msln	1.28	3.71E-01	Clec4a2	1.26	6.67E-02	Tgfb1	1.12	8.78E-01	Slc7a11	1.16	9.34E-01
Msr1	1.29	3.06E-01	Cspg4	1.28	1.47E-01	Nfkb2	1.26	2.87E-03	Pla2g1b	1.12	9.90E-01	Il7	1.16	7.82E-01
Il12b	1.29	6.43E-01	Il12b	1.28	4.34E-01	Ccl20	1.26	4.48E-01	Il21	1.11	9.90E-01	Cd160	1.15	8.63E-01
Lcn2	1.29	6.21E-01	Tyk2	1.27	1.23E-01	Oasl1	1.26	2.04E-01	Angpt1	1.11	8.96E-01	Il22ra2	1.15	8.85E-01
Ifng	1.28	6.73E-01	Psmb10	1.27	3.83E-02	Batf	1.25	1.43E-01	Ifna4	1.11	9.90E-01	C8g	1.15	9.20E-01
Tnfrsf1b	1.28	1.48E-01	Cfp	1.27	2.70E-01	Usp18	1.25	4.91E-02	Vegfc	1.11	9.21E-01	Cd1d2	1.15	8.42E-01
Tap2	1.28	2.77E-01	Il10ra	1.27	5.43E-02	Card9	1.25	2.14E-01	Thbd	1.11	9.90E-01	Dll4	1.15	6.25E-01
Ifit1	1.28	4.22E-01	Tnfaip3	1.27	1.74E-01	Klra4	1.24	4.75E-01	Mr1	1.11	8.78E-01	Il1rl2	1.15	7.90E-01
Itgam	1.28	4.15E-01	Prg2	1.27	6.18E-01	Nos2	1.24	1.02E-01	Itgb4	1.11	9.90E-01	Rora	1.15	6.61E-01
Mcam	1.27	1.44E-01	Ccl1	1.26	5.28E-01	Psmb10	1.24	1.37E-02	Ifitm2	1.11	8.78E-01	Il34	1.15	5.42E-01
Vcam1	1.27	3.14E-01	Rsad2	1.26	4.13E-01	Tnfaip3	1.24	8.27E-02	Trem1	1.11	9.90E-01	Ctsh	1.15	7.25E-01
Card9	1.26	5.77E-01	Tnfsf4	1.26	5.43E-01	Dmbt1	1.24	7.29E-01	Isg20	1.11	8.78E-01	Il6ra	1.15	6.11E-01
Entpd1	1.26	5.03E-02	Cxcl3	1.25	6.44E-01	Il10ra	1.24	2.33E-02	H2-Q1	1.11	9.90E-01	Mif	1.15	8.40E-01
C2	1.26	6.32E-01	Abca1	1.25	9.85E-02	Il4	1.24	4.63E-01	Il19	1.11	9.90E-01	Bst1	1.15	7.82E-01
Sbno2	1.25	5.42E-02	Cd276	1.25	7.89E-02	Ifna1	1.24	4.63E-01	Snai1	1.10	9.90E-01	Cebpb	1.15	8.42E-01
Ptgdr2	1.25	5.66E-01	Ccl24	1.25	6.43E-01	Trem1	1.23	3.44E-01	Zfp13	1.10	9.72E-01	Ikzf1	1.15	9.34E-01
Zfp13	1.24	3.38E-01	Cxcl9	1.25	5.34E-01	Csf2rb	1.23	1.06E-02	Csf2rb	1.10	8.96E-01	Creb1	1.14	3.42E-01
Ifna1	1.24	7.69E-01	Irf7	1.25	3.52E-01	Klra2	1.22	2.23E-01	Tnfsf12	1.10	9.48E-01	Clec4a2	1.14	7.25E-01
Itgb3	1.24	4.59E-01	H2-Q1	1.25	5.83E-01	Cklf	1.22	2.64E-01	Sh2d1b1	1.10	9.90E-01	Jun	1.14	7.90E-01
Pvr	1.23	1.30E-01	Tirap	1.24	8.42E-02	Ido1	1.22	4.75E-01	Rora	1.10	9.73E-01	Foxj1	1.14	8.63E-01
Emr1	1.23	3.85E-01	Fcgr4	1.24	3.97E-01	Zfp13	1.22	7.45E-02	Cxcl16	1.10	9.90E-01	F13a1	1.14	8.65E-01
Vim	1.23	1.90E-01	Ptprc	1.24	3.85E-01	Il1b	1.22	4.44E-01	Abca1	1.10	9.46E-01	Il17ra	1.13	7.54E-01
Vhl	1.23	2.49E-01	Gpr183	1.24	3.00E-01	Irf7	1.22	2.64E-01	Csf3	1.10	9.90E-01	Pvr	1.13	4.88E-01
Cd2	0.63	3.94E-01	Cd5	0.67	6.53E-01	Cd40	0.67	6.05E-04	Cd3g	0.77	9.90E-01	Klrg1	0.76	4.97E-01
Defb1	0.62	8.62E-01	Fos	0.67	5.50E-01	Cd53	0.66	2.92E-02	Ecsit	0.76	8.78E-01	Klrd1	0.76	3.42E-01
Slamf6	0.62	4.37E-01	Cr2	0.66	1.36E-02	Ccl27a	0.66	2.51E-03	Osm	0.76	8.78E-01	H2-Ab1	0.76	3.42E-01
Klrc2	0.62	9.39E-02	Atg16l1	0.66	5.98E-04	Slamf1	0.66	1.02E-01	Rel	0.76	2.11E-01	Il18rap	0.75	3.26E-01
Cd53	0.60	1.48E-01	H2-DMb2	0.66	3.83E-02	Ly9	0.66	1.04E-01	Ly9	0.76	8.96E-01	Tlr2	0.75	1.19E-01
Cd8b1	0.60	8.34E-01	Chit1	0.66	7.82E-01	Serpinb2	0.65	5.03E-01	Gzmm	0.75	8.78E-01	Chit1	0.75	9.78E-01
Il9	0.60	3.33E-01	Cd36	0.65	3.27E-02	Cd37	0.65	2.59E-03	Ccl25	0.75	9.90E-01	H2-Q2	0.75	4.94E-01

Mme	0.59	1.25E-01	Defb1	0.65	7.70E-01	Cxcl14	0.65	2.44E-02	Il15ra	0.74	8.78E-01	Gpr183	0.75	3.26E-01
Cxcr5	0.59	1.42E-01	H2-Ob	0.65	2.78E-02	Cmah	0.64	2.70E-03	Il2	0.74	8.78E-01	Tnfsf10	0.74	3.38E-01
Tnfrsf4	0.58	2.71E-01	Cd9	0.63	1.55E-02	Flt3l	0.64	1.50E-04	Ifi27	0.74	9.03E-01	C3ar1	0.74	2.17E-01
Ly9	0.58	3.11E-01	Cxcl14	0.63	7.12E-02	Defb1	0.64	6.61E-01	C8b	0.74	8.78E-01	Ccr7	0.74	2.46E-01
Mef2c	0.58	1.48E-01	Cmah	0.62	1.21E-02	Ltb	0.63	2.18E-02	Cd27	0.74	9.90E-01	Fcer2a	0.74	5.04E-01
Cr2	0.58	1.27E-02	Slamf6	0.61	2.00E-01	Il16	0.63	2.82E-02	Cd2	0.73	8.78E-01	Il1b	0.73	6.11E-01
Cd37	0.58	2.81E-02	C8g	0.61	2.28E-01	Cr2	0.62	3.33E-04	Runx3	0.72	8.78E-01	Il3	0.72	7.54E-01
Cfh	0.57	1.46E-02	Arg1	0.60	6.04E-01	Slamf6	0.62	8.45E-02	Il1b	0.72	8.78E-01	Fcgr1	0.72	8.33E-02
Sell	0.57	6.12E-02	Pparg	0.60	3.69E-01	Cd3e	0.62	4.40E-01	Ccl20	0.72	8.96E-01	Nos2	0.72	2.17E-01
Twist1	0.56	7.10E-01	Cfh	0.59	2.78E-03	Cd1d1	0.62	1.44E-01	Tnfrsf4	0.71	8.78E-01	Tnfrsf12a	0.72	1.35E-01
Ltb	0.56	1.30E-01	Cd22	0.59	6.83E-03	Ccr7	0.62	3.34E-04	Egr3	0.71	8.78E-01	Xcr1	0.72	6.27E-01
Cd3g	0.56	7.17E-01	Xcr1	0.58	7.81E-02	Itga2b	0.61	9.23E-02	Ccr7	0.71	5.74E-01	Ido1	0.71	6.11E-01
Spink5	0.55	7.06E-01	Sh2b2	0.58	3.79E-01	Cd79a	0.61	3.72E-02	Masp2	0.71	6.83E-01	Ccl12	0.71	3.42E-01
Il16	0.55	1.40E-01	Cd19	0.58	1.21E-02	H2-DMb2	0.60	1.48E-03	Il17f	0.71	8.78E-01	Cd7	0.70	2.17E-01
Ccr7	0.55	8.55E-03	Mef2c	0.57	4.03E-02	Masp2	0.60	7.69E-04	Thy1	0.70	9.90E-01	Tnfrsf13c	0.70	4.88E-01
Arg1	0.55	6.97E-01	Jun	0.57	4.64E-03	Cfh	0.58	1.37E-04	Saa1	0.70	9.46E-01	Ciita	0.69	2.17E-01
Cd27	0.55	7.06E-01	Mme	0.57	1.72E-02	Mme	0.58	3.15E-03	Slamf6	0.70	8.78E-01	Hamp	0.69	9.04E-01
H2-DMb2	0.55	2.80E-02	Flt3l	0.57	3.72E-04	Mef2c	0.58	7.93E-03	Il23a	0.70	8.78E-01	Ccr2	0.69	8.15E-03
Cd79a	0.55	1.77E-01	Il1r2	0.56	3.90E-01	Arg1	0.58	3.97E-01	Tnfrsf10b	0.70	5.74E-01	Gzma	0.69	2.73E-01
Cd22	0.54	1.67E-02	Btla	0.54	2.69E-03	Cd22	0.56	2.69E-04	Mnx1	0.68	8.78E-01	Cr2	0.68	1.22E-01
Cd3e	0.54	6.94E-01	Egr3	0.54	8.87E-02	H2-Ob	0.56	2.89E-04	H60a	0.68	8.78E-01	Cxcl3	0.67	6.25E-01
Cd70	0.53	3.99E-01	Masp2	0.54	1.90E-03	Spink5	0.55	3.60E-01	Lck	0.67	9.90E-01	Pou2f2	0.65	1.03E-01
Tnfrsf13c	0.53	1.01E-01	Spink5	0.54	5.52E-01	Chit1	0.54	5.22E-01	Timd4	0.67	8.78E-01	Ms4a1	0.65	1.75E-01
Il5ra	0.53	1.33E-01	C7	0.53	1.27E-02	Blk	0.54	8.32E-04	Cd4	0.67	9.90E-01	Ccl7	0.65	1.72E-01
Serpinb2	0.50	6.05E-01	Il12a	0.51	2.54E-03	Cxcr5	0.53	1.84E-03	Cd69	0.66	8.78E-01	Ccr5	0.64	8.05E-02
Il12a	0.50	1.17E-02	Slamf1	0.50	5.25E-02	Il1r2	0.52	2.02E-01	Cd3e	0.66	9.90E-01	Cd180	0.64	2.75E-02
Il1r2	0.49	5.64E-01	Pax5	0.50	3.46E-03	Cd5	0.52	2.67E-01	Mpo	0.66	9.10E-01	Klra1	0.64	5.11E-01
H2-Ob	0.48	6.03E-03	Itgae	0.49	1.06E-03	Ppbp	0.52	3.87E-02	Cd8b1	0.64	9.90E-01	Cd19	0.63	1.75E-01
Itgae	0.48	6.97E-03	Cxcr5	0.48	7.19E-03	Twist1	0.51	2.97E-01	Rag1	0.64	9.90E-01	H2-Ob	0.63	1.22E-01
C7	0.48	2.97E-02	Ada	0.48	8.06E-01	Il12a	0.50	1.08E-04	Pparg	0.64	8.96E-01	Ncr1	0.63	6.55E-02
Chit1	0.45	7.11E-01	Mpped1	0.47	4.27E-01	C7	0.50	6.38E-04	Cd6	0.63	9.46E-01	Ccl4	0.62	3.36E-01
Blk	0.43	7.78E-03	Twist1	0.46	4.34E-01	Xcr1	0.49	3.20E-03	Sele	0.62	8.78E-01	Il6	0.62	4.53E-01
Pax5	0.41	6.03E-03	Cd1d1	0.45	6.15E-02	Itgae	0.49	4.28E-05	Ccr3	0.61	9.90E-01	Ccl5	0.62	6.55E-02
Xcr1	0.41	3.91E-02	Itga2b	0.45	4.98E-02	Cd19	0.49	1.06E-04	Cd8a	0.60	9.90E-01	C8b	0.61	3.26E-01
Cd5	0.41	4.80E-01	A2m	0.44	6.42E-02	Pax5	0.45	8.41E-05	Cd1d1	0.60	8.78E-01	S100a8	0.60	1.91E-01
Cd19	0.41	3.69E-03	Cd207	0.44	1.79E-03	Btla	0.43	5.57E-06	Cfd	0.60	9.90E-01	C8a	0.58	8.15E-01
Timd4	0.39	1.44E-01	Cd79b	0.44	1.81E-05	Timd4	0.41	1.12E-02	S100b	0.57	9.18E-01	Cxcl5	0.58	1.38E-01
Btla	0.34	2.96E-04	Ms4a1	0.44	1.91E-04	Cd207	0.39	2.32E-05	C8g	0.55	8.78E-01	Lif	0.57	2.61E-04
Cd207	0.34	3.06E-03	Timd4	0.43	7.71E-02	Cd79b	0.39	9.29E-08	Ccr9	0.55	9.90E-01	Rrad	0.57	2.75E-02
Cd79b	0.34	9.03E-05	Fcer2a	0.41	2.72E-04	Fcer2a	0.37	3.81E-06	Ccl26	0.51	5.74E-01	Prf1	0.56	2.75E-02
Fcer2a	0.34	1.17E-03	Ppbp	0.38	3.27E-02	Ms4a1	0.37	5.81E-07	Fos	0.43	8.78E-01	Cx3cr1	0.53	6.94E-04
Ms4a1	0.31	1.38E-04	Glycam1	0.37	2.41E-01	Cfd	0.31	3.67E-01	Glycam1	0.41	8.78E-01	Cxcl10	0.48	6.55E-02
Ada	0.17	6.75E-01	Cfd	0.15	3.39E-01	Ada	0.28	4.93E-01	Sh2b2	0.33	8.78E-01	Ccl2	0.47	8.15E-03

Table S2: Top 150 & bottom 50 NanoString PanCancer Pathways panel hits ranked by fold change (FC). Related to Figures 2&7

Fig. 2B Vehicle vs Chemo			Fig. 7B Vehicle vs Chemo			Fig. 2B+&B Veh. vs Chemo			Fig. 7C Vehicle ± Nav			Fig. 7C Chemo ± Nav		
Factor	FC	adj.P.Val	Factor	FC	adj.P.Val	Factor	FC	adj.P.Val	Factor	FC	adj.P.Val	Factor	FC	adj.P.Val
Cdkn1a	4.96	7.97E-05	Cdkn1a	3.98	4.10E-05	Cdkn1a	4.44	2.67E-07	Zbtb16	5.38	7.21E-02	Zbtb16	9.45	2.95E-03
Mmp3	4.02	2.32E-02	Tnc	3.53	1.96E-03	Tnc	3.68	5.60E-05	Ifnb1	3.33	8.02E-01	Wee1	1.83	2.95E-03
Tnc	3.85	1.04E-02	Lif	2.81	4.10E-05	Mmp3	3.13	9.88E-04	Nodal	2.29	8.02E-01	Bcl2l1	1.73	2.95E-03
Ifnb1	3.33	3.61E-01	Pla1a	2.52	7.45E-02	Lif	2.58	2.62E-06	Gata1	2.27	1.14E-01	Fgf3	1.66	7.92E-01
Ccna2	2.81	4.87E-01	Mmp3	2.44	3.47E-02	Ifnb1	2.41	1.32E-01	Rasgrf1	1.95	8.02E-01	Sox9	1.63	4.91E-01
Pla2g5	2.66	2.11E-01	Etv4	2.36	1.40E-02	Il6	2.35	3.67E-03	Mpl	1.79	8.02E-01	Comp	1.62	8.24E-01
Nr4a1	2.63	1.16E-01	Il6	2.26	3.05E-02	Ccna2	2.27	1.36E-01	Wnt10a	1.78	8.02E-01	Ddb2	1.60	2.40E-01
Il6	2.45	1.19E-01	Pax3	2.21	2.40E-02	Brip1	2.05	8.17E-03	Dll3	1.72	8.04E-01	Alk	1.58	8.68E-01
Lif	2.37	4.65E-03	Hist1h3b	2.15	2.75E-01	Hist1h3b	2.04	1.73E-01	Pitx2	1.63	8.02E-01	Figf	1.57	1.05E-01
Ttk	2.33	5.38E-01	Cxcl5	2.09	7.84E-03	Col5a1	2.03	5.51E-05	Creb3l3	1.62	8.04E-01	Ccnb1	1.54	7.24E-01
Inhba	2.32	8.22E-01	Wnt3a	2.06	1.96E-03	Etv4	2.00	6.29E-03	Il19	1.61	8.02E-01	Hspa1a	1.54	9.24E-01
Nr4a3	2.31	1.71E-01	Col5a1	2.01	1.96E-03	Thbs1	1.97	2.15E-03	Amh	1.60	8.02E-01	Fst	1.53	6.31E-01
Thbs1	2.25	4.96E-02	Brip1	2.01	5.47E-02	Ttk	1.93	1.90E-01	Fgf20	1.59	8.02E-01	Map3k12	1.53	4.91E-01
Top2a	2.18	7.04E-01	Fgf23	1.99	2.53E-01	Inhba	1.90	4.64E-01	Hnf1a	1.57	8.02E-01	Fgf20	1.52	7.14E-01
Brip1	2.10	2.01E-01	Gadd45g	1.98	2.00E-02	Top2a	1.89	2.96E-01	Ccnb1	1.56	8.02E-01	Ccne2	1.52	8.68E-01
Cxxc4	2.05	1.38E-01	Nodal	1.93	3.05E-01	Pla1a	1.84	1.26E-01	Rps27a	1.54	8.04E-01	Cacna1h	1.51	7.92E-01
Col5a1	2.05	1.04E-02	Pgf	1.88	1.16E-03	Dll3	1.83	1.90E-01	Pla2g4c	1.54	8.02E-01	Fgf14	1.48	9.09E-01
Col24a1	2.03	2.85E-01	Csf3r	1.87	2.99E-02	Nodal	1.77	2.51E-01	Grin2a	1.53	8.02E-01	Cntfr	1.46	6.94E-01
Lama1	1.97	2.40E-01	Cxcl1	1.86	5.72E-02	Igf1	1.75	1.09E-02	Nog	1.52	8.79E-01	Epo	1.46	8.68E-01
Igf1	1.95	1.19E-01	Ccna2	1.84	4.15E-01	Gdf6	1.71	3.76E-02	Prmt8	1.50	8.02E-01	Pla2g5	1.46	8.88E-01
Hist1h3b	1.95	7.29E-01	Nog	1.83	3.86E-01	Lama1	1.71	4.32E-02	Birc7	1.49	8.02E-01	Il2ra	1.45	9.09E-01
Dll3	1.85	7.04E-01	Dll3	1.81	3.25E-01	Cxcl1	1.70	3.33E-02	Gng4	1.49	8.02E-01	Ptcra	1.45	9.41E-01
Chad	1.84	5.55E-01	Gdf6	1.77	9.06E-02	Pla2g4c	1.67	5.16E-02	Alk	1.48	8.18E-01	Mpl	1.44	8.38E-01
Cdkn2a	1.82	2.06E-01	Ppp2r2c	1.74	1.01E-01	Col27a1	1.65	3.64E-03	Sfrp2	1.47	8.02E-01	Il4	1.43	8.42E-01
Col2a1	1.79	6.28E-01	Sfrp2	1.74	1.04E-01	Pax3	1.65	6.03E-02	Ppp3r2	1.44	8.02E-01	Il1r2	1.43	9.31E-01
Il11	1.78	4.17E-01	Pla2g4c	1.74	1.15E-01	Flna	1.64	4.90E-04	Hoxa10	1.42	8.04E-01	Il22ra2	1.42	8.38E-01
Wnt5a	1.77	1.09E-03	Ifnb1	1.74	5.12E-01	Smc1b	1.62	8.29E-02	Pla2g10	1.42	9.32E-01	Hist2h3b	1.42	9.31E-01
Wnt11	1.75	5.24E-02	Thbs1	1.72	5.01E-02	Pgf	1.62	6.45E-04	Wnt3a	1.42	8.02E-01	Fzd10	1.40	9.31E-01
Ifna1	1.72	5.55E-01	Pdgfrb	1.71	4.95E-03	Rad51	1.61	1.93E-01	Tnr	1.40	8.04E-01	Ttk	1.39	9.31E-01
Etv4	1.70	3.61E-01	Pla2g2a	1.71	2.92E-02	Cdc25c	1.61	3.15E-01	Lefty2	1.40	8.08E-01	Ezh2	1.39	8.68E-01
Ptcra	1.69	8.71E-01	Rad51	1.71	2.81E-01	Cxcl5	1.59	2.94E-02	Pax3	1.40	8.04E-01	Il11ra1	1.39	7.92E-01
Fgf8	1.67	4.98E-01	Cdc25c	1.71	4.05E-01	Il23a	1.59	3.56E-02	Il2	1.39	8.08E-01	Igfbp3	1.38	8.05E-01
Col27a1	1.66	1.19E-01	Flna	1.70	4.58E-03	Epha2	1.58	3.76E-03	Wt1	1.37	8.02E-01	Gria3	1.38	4.91E-01
Il23a	1.65	3.28E-01	Il2rb	1.70	8.31E-03	Wnt3a	1.58	4.19E-03	Tnn	1.37	8.02E-01	Rad51	1.37	8.88E-01
Smc1b	1.65	5.10E-01	Fzd2	1.68	5.10E-03	Fgf8	1.56	1.04E-01	Wnt3	1.36	8.04E-01	Gngt1	1.36	8.68E-01
Gdf6	1.65	4.65E-01	Epha2	1.66	1.73E-02	Wnt5a	1.56	3.94E-05	Casp9	1.36	5.75E-01	Brca2	1.36	7.39E-01
Spry4	1.64	4.96E-02	Il13	1.66	2.69E-01	Sfrp2	1.56	8.37E-02	Fgf15	1.36	8.04E-01	Cxxc4	1.36	7.94E-01
Nodal	1.63	8.19E-01	Plau	1.65	2.82E-04	Col5a2	1.56	1.08E-02	Ntrk1	1.35	8.02E-01	Cdk2	1.36	4.91E-01
Col5a2	1.61	1.98E-01	Creb3l3	1.64	4.13E-01	Wnt11	1.55	4.19E-03	Zic2	1.35	8.17E-01	Mmp7	1.36	8.68E-01
E2f1	1.61	2.02E-01	Col27a1	1.64	2.59E-02	Fzd2	1.54	1.91E-03	Cacng6	1.34	9.45E-01	Rps6ka6	1.35	8.42E-01
Pla2g4c	1.60	5.38E-01	Top2a	1.63	5.56E-01	Col24a1	1.53	1.55E-01	Cacng4	1.33	8.12E-01	Lef1	1.35	9.09E-01
Socs3	1.60	9.79E-02	Wnt7a	1.63	1.10E-02	Socs3	1.52	3.67E-03	Smc1b	1.33	8.17E-01	Hist1h3b	1.35	9.43E-01
Jak3	1.59	1.04E-02	Lama5	1.62	2.59E-02	Prmt8	1.51	6.54E-02	Wnt2b	1.33	8.04E-01	Fas	1.34	3.81E-01
Flna	1.59	7.05E-02	Grin2a	1.62	9.60E-02	Pdgfrb	1.51	3.64E-03	Lama3	1.33	8.02E-01	Myb	1.34	9.31E-01
Tnfrsf10b	1.58	3.89E-02	Notch3	1.62	4.67E-03	Epor	1.51	3.64E-03	Pla2g4e	1.33	9.55E-01	Fgf13	1.34	9.41E-01
Col1a1	1.58	9.93E-02	Epor	1.60	9.94E-03	Il11	1.51	1.58E-01	Efna3	1.32	9.45E-01	Klf4	1.34	7.35E-01
Cacna1h	1.58	6.74E-01	Smc1b	1.59	2.20E-01	Brca1	1.51	3.30E-01	Gdf6	1.32	8.08E-01	Prmt8	1.33	7.92E-01
Fos	1.58	8.22E-01	Inhbb	1.59	2.65E-01	Bax	1.48	2.10E-04	Wnt1	1.32	8.79E-01	Cacng6	1.33	9.45E-01
Ccne2	1.57	7.78E-01	Ttk	1.59	5.12E-01	Il3ra	1.48	1.91E-03	Flt1	1.31	8.02E-01	Stat4	1.33	6.94E-01
Bax	1.57	1.19E-02	Fn1	1.58	1.36E-02	Plau	1.47	1.73E-04	Fzd9	1.31	9.26E-01	Cdc25c	1.33	9.31E-01
Cxcl1	1.56	5.38E-01	Igf1	1.58	1.26E-01	Ddit4	1.46	2.32E-02	Calml3	1.31	9.78E-01	Cdc7	1.32	8.68E-01
Prom1	1.56	3.31E-01	Il3ra	1.58	6.51E-03	Hist2h3b	1.46	5.10E-01	Cacnb4	1.31	8.04E-01	Ccnb3	1.32	9.21E-01
Chek2	1.54	3.61E-01	Fgf15	1.57	2.08E-01	Chad	1.46	3.43E-01	Nfe2l2	1.31	8.02E-01	Wnt2b	1.32	8.73E-01
Myb	1.54	8.41E-01	Inhba	1.55	7.26E-01	Jak3	1.45	4.90E-04	Bcl2a1a	1.31	8.02E-01	Rasal1	1.31	8.68E-01
Brca1	1.53	8.05E-01	Prmt8	1.54	1.58E-01	Cdkn2a	1.44	1.05E-01	Hoxa11	1.29	9.45E-01	Mcm7	1.31	8.68E-01
Rad51	1.52	7.60E-01	Fgf20	1.54	2.91E-01	Crlf2	1.43	1.88E-04	Cacng1	1.29	9.66E-01	Ccno	1.31	4.91E-01
Cdc25c	1.51	8.41E-01	Il23a	1.53	1.30E-01	Il2rb	1.43	2.17E-02	Il20rb	1.29	9.27E-01	Il20rb	1.31	9.31E-01
Col6a6	1.51	7.60E-01	Lama3	1.52	2.71E-02	Dtx4	1.42	3.64E-03	Gzmb	1.29	8.04E-01	Col4a6	1.30	8.06E-01
Fgf17	1.51	7.37E-01	Col5a2	1.51	7.72E-02	Nupr1	1.42	1.55E-02	Wee1	1.28	8.02E-01	Arid2	1.29	8.68E-01
Epha2	1.51	2.03E-01	Wt1	1.51	2.07E-01	Cd14	1.42	1.02E-03	Ret	1.28	8.30E-01	Rps6ka5	1.29	5.72E-01
Ptprr	1.50	1.92E-01	Creb3l1	1.51	7.45E-02	Tnfrsf10b	1.42	3.67E-03	Wnt7b	1.28	9.45E-01	H2afx	1.29	9.10E-01
Prmt8	1.49	5.38E-01	Cdkn1c	1.50	4.67E-03	Mdm2	1.41	2.09E-04	Rac3	1.28	8.02E-01	Lamc3	1.28	8.85E-01
Chek1	1.49	7.66E-01	Csf1r	1.50	7.53E-03	Fgf15	1.41	2.17E-01	Bambi	1.27	8.02E-01	Whsc1	1.28	8.45E-01
Shc3	1.49	7.67E-01	Hist2h3b	1.50	6.16E-01	Col2a1	1.41	3.73E-01	Csf2rb	1.27	8.02E-01	Il11ra2	1.28	9.31E-01
Ddit4	1.49	2.71E-01	Smo	1.49	3.49E-02	Fgf23	1.40	4.92E-01	Gadd45g	1.26	8.17E-01	Fgfr4	1.28	8.68E-01
Fosl1	1.48	4.87E-01	Brca1	1.49	4.74E-01	Col1a2	1.40	1.04E-02	Ifng	1.26	9.15E-01	Birc7	1.27	8.77E-01
Nupr1	1.48	1.98E-01	Crlf2	1.48	1.96E-03	Lama5	1.40	3.69E-02	Wnt4	1.25	9.32E-01	Notch1	1.27	4.91E-01
Casp12	1.48	5.59E-02	Lama1	1.48	2.77E-01	Gadd45g	1.40	1.35E-01	Efna1	1.25	8.08E-01	Bnip3	1.27	8.88E-01
Ccna1	1.47	6.74E-01	Birc7	1.47	2.55E-01	Csf1r	1.40	3.64E-03	Ccnb3	1.25	9.27E-01	Baiap3	1.27	7.39E-01
Fzd9	1.46	8.41E-01	Cacng4	1.47	3.05E-01	Csf3	1.40	4.78E-01	Dll1	1.25	8.02E-01	Ret	1.26	9.09E-01
Shc2	1.44	5.38E-01	Col4a4	1.46	2.26E-02	Fanca	1.39	4.48E-02	Fzd2	1.25	8.02E-01	Chek1	1.26	9.31E-01
Ccne1	1.43	8.41E-01	Fgf8	1.46	2.93E-01	Fen1	1.39	1.03E-01	Itgb4	1.25	9.04E-01	Col11a2	1.26	9.31E-01
Rasgrf1	1.43	8.27E-01	Ppp3r2	1.45	3.31E-01	Ifna1	1.38	3.31E-01	Fzd10	1.24	9.45E-01	Col11a1	1.26	9.31E-01
Epor	1.42	2.23E-01	Csf2rb	1.45	1.73E-02	Ppp3r2	1.38	2.58E-01	Smo	1.24	8.02E-01	Ets2	1.26	6.00E-01
Hist2h3b	1.42	9.00E-01	Hist2h3c2	1.45	1.60E-02	Col4a4	1.38	1.06E-02	Il3ra	1.24	8.02E-01	Stmn1	1.26	8.68E-01
H2afx	1.42	7.86E-01	Itgb6	1.44	1.04E-01	Csf3r	1.37	1.54E-01	Creb5	1.24	8.02E-01	Brca1	1.25	9.31E-01

Arnt2	1.42	4.87E-01	Ddit4	1.44	1.08E-01	Rps27a	1.37	4.59E-01	Il11	1.23	9.04E-01	Pkmyt1	1.25	9.02E-01
Fanca	1.42	3.61E-01	Socs3	1.44	4.67E-02	Wnt7a	1.37	3.70E-02	Pias1	1.23	8.02E-01	Pdgfra	1.25	8.06E-01
Mdm2	1.41	3.05E-02	Tpo	1.43	2.82E-01	Cacng4	1.37	3.06E-01	Csf2	1.23	8.17E-01	Socs2	1.25	7.39E-01
Dtx4	1.41	1.45E-01	Fen1	1.43	1.82E-01	E2f1	1.36	6.86E-02	Dkk4	1.22	8.18E-01	Top2a	1.25	9.45E-01
Reln	1.41	6.74E-01	Dtx4	1.43	1.93E-02	Lama3	1.36	3.26E-02	Itgb3	1.22	8.02E-01	Ccne1	1.25	9.31E-01
Col3a1	1.41	5.44E-01	Cd14	1.43	7.24E-03	Col1a1	1.36	2.81E-02	Fgfr2	1.22	8.02E-01	Dkk1	1.25	9.02E-01
Cd14	1.41	8.93E-02	Gzmb	1.42	2.13E-01	Chek1	1.36	4.08E-01	Cebpe	1.22	9.15E-01	Creb3l3	1.25	9.45E-01
Mcm5	1.41	7.60E-01	Rps27a	1.42	5.12E-01	Casp12	1.36	4.81E-03	Mapk8ip1	1.22	8.02E-01	Dnmt1	1.24	7.92E-01
Fzd2	1.40	2.11E-01	Casp9	1.42	1.93E-02	Chek2	1.35	1.42E-01	Il13ra2	1.22	9.45E-01	Srsf2	1.24	3.35E-01
Col1a2	1.40	2.22E-01	Ngfr	1.42	2.75E-01	Fosl1	1.35	1.58E-01	Smad2	1.21	8.02E-01	Id1	1.24	5.50E-01
Sfrp2	1.40	7.29E-01	Hoxa10	1.42	4.15E-01	H2afx	1.35	3.81E-01	Pdgfra	1.21	8.12E-01	Il23r	1.24	8.68E-01
Il3ra	1.39	1.88E-01	Csf2	1.42	2.08E-01	Itgb6	1.35	7.84E-02	Prkar1b	1.21	8.02E-01	Amh	1.24	9.34E-01
Pgf	1.39	2.41E-01	Mdm2	1.41	4.21E-03	Rasgrf1	1.35	4.66E-01	Fgf17	1.21	9.32E-01	Npm1	1.23	8.38E-01
Cdc6	1.39	8.15E-01	Zic2	1.41	4.13E-01	Wt1	1.34	2.37E-01	Kit	1.21	8.08E-01	Ccna2	1.23	9.45E-01
Csf3	1.39	8.78E-01	Bax	1.41	8.66E-03	Shc4	1.34	2.68E-01	Rad51	1.21	9.45E-01	Il7	1.23	8.68E-01
Crlf2	1.39	4.96E-02	Hoxa11	1.41	6.42E-01	Fn1	1.34	3.56E-02	Ccna1	1.21	9.04E-01	Six1	1.22	9.45E-01
Vegfc	1.38	1.52E-01	Csf3	1.41	6.03E-01	Notch3	1.33	1.73E-02	Hhip	1.20	8.02E-01	Il10	1.22	9.31E-01
Ccnd1	1.38	4.36E-01	Fzd8	1.40	1.57E-01	Nr4a3	1.33	3.59E-01	Il12b	1.20	9.32E-01	Acvr1c	1.22	9.45E-01
Dll1	1.38	2.03E-01	Hmga1	1.40	2.77E-01	Myb	1.33	5.72E-01	Idh2	1.20	9.04E-01	Itga8	1.22	8.68E-01
Prl	1.37	7.07E-01	Col1a2	1.40	5.47E-02	Prom1	1.33	1.73E-01	Wnt2	1.20	8.30E-01	Fgf22	1.22	9.02E-01
Hnf1a	1.36	8.41E-01	Fasl	1.39	3.13E-01	Pla2g2a	1.32	1.56E-01	Cacna2d2	1.20	9.45E-01	Cacna1c	1.22	8.42E-01
Pla1a	1.34	8.84E-01	Shc4	1.39	3.56E-01	Cdc6	1.32	4.08E-01	Lama5	1.20	8.08E-01	Cdc25a	1.22	8.38E-01
Fen1	1.34	6.74E-01	Ntrk1	1.39	3.01E-01	Ptprr	1.32	5.26E-02	Dusp5	1.19	8.02E-01	Hdac11	1.21	4.91E-01
Creb3l4	1.34	6.53E-01	Wnt5a	1.38	1.10E-02	Inhbb	1.32	4.07E-01	Bap1	1.19	8.04E-01	Sos1	1.21	6.78E-01
Pdgfrb	1.33	3.61E-01	Il13ra2	1.37	6.29E-01	Ccnd1	1.32	8.75E-02	Itga8	1.19	8.37E-01	Med12	1.20	4.91E-01
Il23r	1.33	7.04E-01	Wnt11	1.37	1.15E-01	Hells	1.31	4.36E-01	Pdgfrb	1.19	8.04E-01	Blm	1.20	9.09E-01
Grin2b	1.33	8.05E-01	Fanca	1.37	1.62E-01	Ccne1	1.31	5.07E-01	Polr2j	1.19	8.04E-01	Wnt2	1.20	9.02E-01
Fgf16	1.33	8.57E-01	Cacna2d4	1.36	2.04E-01	Mcm2	1.30	3.43E-01	Ccno	1.19	8.02E-01	Rasgrp2	1.20	4.91E-01
Il3	1.32	8.41E-01	Nupr1	1.36	1.08E-01	Creb3l1	1.30	1.20E-01	Csf3r	1.18	9.04E-01	Acvr2a	1.20	8.68E-01
Epo	1.32	8.78E-01	Mfng	1.36	4.98E-02	Zic2	1.30	4.12E-01	Mfng	1.18	8.02E-01	Pias1	1.20	8.67E-01
Ccno	1.32	3.60E-01	Cebpe	1.35	4.61E-01	Gadd45a	1.30	1.58E-03	Lamc3	1.18	9.24E-01	Fance	1.20	6.16E-01
Rps27a	1.32	8.90E-01	Hells	1.34	5.33E-01	Pkmyt1	1.30	3.51E-01	Ddit3	1.18	8.02E-01	Fancf	1.19	4.91E-01
Plau	1.31	1.45E-01	Gata3	1.34	3.05E-01	Csf2rb	1.29	2.81E-02	Csf1r	1.18	8.02E-01	Ube2t	1.19	9.45E-01
Pkmyt1	1.31	8.14E-01	Ccnb1	1.34	5.12E-01	Jag2	1.29	1.01E-01	Lefty1	1.18	8.02E-01	Lig4	1.19	9.09E-01
Ccnd2	1.31	1.16E-01	Prkcg	1.34	1.18E-01	Hmga1	1.29	2.91E-01	Dnmt3a	1.18	8.04E-01	Dvl2	1.19	5.18E-01
Ppp3r2	1.31	8.21E-01	Wnt10a	1.34	5.66E-01	Cdkn1c	1.29	1.51E-02	Brip1	1.17	9.35E-01	Fbxw7	1.19	6.75E-01
Il20ra	1.31	9.14E-01	Bambi	1.34	5.29E-02	Ccnd2	1.28	3.76E-03	Akt3	1.17	8.02E-01	Il12b	1.19	9.45E-01
Mcm2	1.30	8.15E-01	Hsp90b1	1.34	5.17E-03	Nog	1.28	6.61E-01	Prdm1	1.17	8.02E-01	Msh6	1.19	9.02E-01
Csf1r	1.30	2.85E-01	Tnf	1.34	2.99E-01	Hnf1a	1.28	4.99E-01	Erbb3	1.17	8.02E-01	Atrx	1.19	5.50E-01
Shc4	1.30	8.09E-01	Il24	1.33	6.44E-01	Birc7	1.28	3.50E-01	Csf3	1.17	9.55E-01	Il5ra	1.19	9.45E-01
Col4a4	1.30	3.79E-01	Jag2	1.33	1.82E-01	Ppp2r2c	1.28	4.06E-01	Hdac4	1.17	8.02E-01	Kmt2c	1.19	4.91E-01
Alk	1.29	9.14E-01	Rfc4	1.32	4.13E-01	Il13	1.28	5.09E-01	Pold1	1.17	8.79E-01	Pcna	1.19	9.24E-01
Casp3	1.29	4.97E-01	Jak3	1.32	3.19E-02	Fasl	1.28	3.50E-01	Ptch1	1.17	8.02E-01	Cacna1d	1.19	8.88E-01
Gadd45a	1.29	1.08E-01	Bmp8a	1.32	4.07E-01	Pla2g5	1.28	5.56E-01	Il12rb2	1.17	9.04E-01	Plcb1	1.18	7.48E-01
Il2	1.29	8.62E-01	Bcl2a1a	1.31	2.15E-01	Ptcra	1.27	7.45E-01	Il23a	1.17	9.15E-01	Il6ra	1.18	7.39E-01
Ret	1.29	8.41E-01	Pim1	1.31	4.15E-01	Mfng	1.27	3.79E-02	Ltbp1	1.17	8.02E-01	Lat	1.18	9.56E-01
Pdgfa	1.29	3.28E-01	Gadd45a	1.31	9.80E-03	Fgf17	1.27	4.94E-01	Notch3	1.16	8.04E-01	Rasgrf1	1.18	9.45E-01
Hells	1.28	8.78E-01	Skp2	1.31	4.65E-01	Bambi	1.27	3.26E-02	Bmp6	1.16	8.24E-01	Angpt1	1.18	7.92E-01
Bdnf	1.28	7.04E-01	Il19	1.31	6.44E-01	Fzd8	1.26	2.08E-01	Cdk4	1.16	8.04E-01	Gli1	1.18	8.42E-01
Blm	1.28	7.60E-01	Mpo	1.30	5.04E-01	Smo	1.26	9.71E-02	Figf	1.16	8.12E-01	Mcm2	1.17	9.31E-01
Plat	1.28	3.60E-01	Mcm2	1.30	4.99E-01	Plat	1.26	3.96E-02	Creb3l1	1.15	8.79E-01	Eya1	1.17	9.04E-01
Pak3	1.28	8.57E-01	Trp53	1.30	6.25E-02	Epo	1.26	5.66E-01	Dll4	1.15	8.17E-01	Cacng1	1.17	9.80E-01
Fgf9	1.27	7.70E-01	Mapk8ip1	1.29	2.04E-01	Casp3	1.26	9.51E-02	Igf1	1.15	9.32E-01	Smc1a	1.17	5.69E-01
Pik3r3	1.27	3.31E-01	Ngf	1.29	6.35E-01	Nr4a1	1.26	4.83E-01	Lamb3	1.15	8.04E-01	Pik3r5	1.17	9.02E-01
Cacng4	1.27	8.62E-01	H2afx	1.28	6.09E-01	Pdgfa	1.26	3.82E-02	Itga6	1.15	8.02E-01	Prkar2b	1.16	9.56E-01
Fgf15	1.27	8.41E-01	Pkmyt1	1.28	5.12E-01	Dll1	1.26	5.26E-02	Igf1r	1.15	8.04E-01	Cdc6	1.16	9.45E-01
Hhip	1.27	5.57E-01	Il11	1.28	5.56E-01	Spry4	1.26	8.13E-02	Gadd45b	1.15	8.04E-01	Nf1	1.16	4.91E-01
Jag2	1.26	6.74E-01	Socs1	1.27	6.11E-01	Rfc4	1.26	3.81E-01	Eya1	1.15	8.92E-01	Il12rb2	1.16	9.29E-01
Itgb6	1.26	7.04E-01	Rasgrf1	1.27	7.09E-01	Arnt2	1.26	2.43E-01	Inhbb	1.15	9.45E-01	Idh2	1.16	9.31E-01
Col11a2	1.25	9.14E-01	Tnfrsf10b	1.27	1.30E-01	Gata3	1.25	3.15E-01	Acvr1b	1.15	8.02E-01	Fgfr3	1.16	8.57E-01
Itgb3	1.24	5.72E-01	Bmp6	1.27	2.82E-01	Vegfc	1.24	4.59E-02	Nkd1	1.15	8.08E-01	Wnt7b	1.16	9.66E-01
Lamc2	1.24	6.97E-01	Spry1	1.26	2.13E-01	Tnfaip3	1.24	1.17E-01	Pold4	1.14	8.02E-01	Plcb4	1.15	6.20E-01
Pax3	1.23	8.78E-01	Sgk2	1.26	8.38E-01	Il13ra2	1.24	6.62E-01	Bmp7	1.14	9.27E-01	Ddit4	1.15	9.02E-01
Fgf18	1.23	7.04E-01	Ccnd1	1.26	2.95E-01	Hist2h3c2	1.24	5.59E-02	Cacnb3	1.14	8.08E-01	Kmt2d	1.15	6.94E-01
Tnfaip3	1.22	6.74E-01	Ccnd2	1.26	3.92E-02	Il19	1.24	6.10E-01	Epo	1.14	9.55E-01	Dtx3	1.15	7.39E-01
Gpc4	1.22	6.73E-01	Tnfaip3	1.26	2.13E-01	Ccne2	1.24	6.40E-01	Lamc2	1.14	8.64E-01	Endog	1.15	9.21E-01
Numbl	1.22	3.28E-01	Gata1	1.26	5.12E-01	Ngfr	1.23	4.08E-01	Fzd8	1.14	9.15E-01	Gata2	1.15	9.02E-01
Col4a3	1.22	7.60E-01	B2m	1.25	9.68E-02	Hoxa11	1.23	6.92E-01	Etv4	1.14	9.45E-01	Sf3b1	1.15	8.38E-01
Lama3	1.22	7.04E-01	Cdc6	1.25	6.44E-01	Trp53	1.23	4.46E-02	Angpt1	1.14	8.08E-01	Hmga2	1.15	9.31E-01
Wnt3a	1.22	7.37E-01	Stk4	1.25	1.35E-01	Hhip	1.23	1.42E-01	Lama1	1.14	9.45E-01	Mpo	1.15	9.45E-01
Wnt3	1.21	8.88E-01	Nkd1	1.25	1.87E-01	Il2	1.23	5.39E-01	Spry1	1.14	8.29E-01	Skp2	1.15	9.43E-01
Lama5	1.21	7.60E-01	Fgf11	1.25	2.76E-01	Prkcg	1.23	1.58E-01	Il1r2	1.14	9.66E-01	Whsc1l1	1.15	7.39E-01
Wee1	1.21	6.01E-01	Casp12	1.24	1.23E-01	Fgf9	1.23	3.67E-01	Plat	1.14	8.04E-01	Mlh1	1.15	8.21E-01
Cxcl5	1.21	8.41E-01	Plat	1.24	1.42E-01	Il24	1.22	6.62E-01	Cdkn1c	1.14	8.04E-01	Tslp	1.14	9.31E-01
Arid2	0.67	6.13E-01	Fgf1	0.69	7.29E-02	Col11a1	0.70	3.39E-01	Col11a1	0.77	9.04E-01	Flnc	0.77	9.45E-01
Wnt6	0.66	6.28E-01	Npm1	0.68	9.06E-02	Ets2	0.70	6.29E-03	Cxxc4	0.77	8.04E-01	Nupr1	0.77	5.50E-01
Prkcb	0.66	7.70E-01	Ccr7	0.67	6.41E-02	Sost	0.69	6.50E-01	Dusp8	0.77	8.02E-01	Fgf18	0.76	5.50E-01
Il20rb	0.66	8.18E-01	Cxxc4	0.67	2.05E-01	Stat4	0.69	4.86E-02	Il23r	0.77	8.02E-01	Fgf6	0.76	8.88E-01
Creb3l3	0.65	8.22E-01	Eif4ebp1	0.67	1.49E-01	Bmp7	0.69	7.79E-02	Tnfrsf10b	0.76	8.02E-01	Col1a1	0.76	4.91E-01
Dtx1	0.65	3.34E-01	Six1	0.67	5.62E-01	Lef1	0.69	3.39E-01	Mapk10	0.76	9.45E-01	Tpo	0.76	8.68E-01
Cd40	0.65	5.24E-02	Mpl	0.66	3.03E-01	Sos1	0.69	1.74E-03	Socs3	0.76	8.02E-01	Pla2g10	0.76	9.45E-01

Egf	0.64	8.62E-01	Thbs4	0.66	5.80E-01	Fgf10	0.68	4.87E-03	Jun	0.76	8.02E-01	Tgfb3	0.76	2.34E-01
Il12b	0.64	7.23E-01	Pla2g3	0.66	6.45E-01	Itga8	0.68	4.09E-02	Acvr1c	0.75	9.27E-01	Cdkn2b	0.76	6.94E-01
Wnt4	0.64	7.60E-01	Fos	0.65	5.04E-01	Jun	0.68	2.32E-02	Lep	0.75	9.04E-01	Ngfr	0.76	8.42E-01
Lefty2	0.63	7.04E-01	Fgf10	0.64	1.11E-02	Il12b	0.68	2.76E-01	Vegfb	0.74	8.02E-01	Ntrk1	0.75	8.42E-01
Col11a1	0.63	7.23E-01	Sost	0.63	6.64E-01	Egf	0.68	5.10E-01	Ifna1	0.74	8.17E-01	Fgf4	0.75	9.09E-01
Rasal1	0.63	5.38E-01	Ets2	0.63	8.46E-03	Il1r2	0.68	5.26E-01	Il6	0.74	8.21E-01	Tlr2	0.75	6.00E-01
Cacna1d	0.63	2.22E-01	Sos1	0.62	3.95E-03	Klf4	0.67	4.09E-02	Prlr	0.74	8.21E-01	Fasl	0.75	8.56E-01
Stat4	0.63	2.85E-01	Bnip3	0.62	1.40E-01	Cacna1d	0.67	2.32E-02	Rasgrp1	0.73	8.92E-01	Pla2g2a	0.75	6.94E-01
Ccnb3	0.62	7.29E-01	Osm	0.62	1.14E-01	Bnip3	0.67	9.43E-02	Il5ra	0.73	8.37E-01	Il2rb	0.75	4.91E-01
Il4	0.62	6.74E-01	Pla2g5	0.61	3.53E-01	Il22ra2	0.66	1.63E-01	Tnf	0.73	8.02E-01	Creb3l1	0.73	5.18E-01
Lef1	0.61	7.04E-01	Fgfr4	0.61	1.01E-01	Pla2g4e	0.66	6.51E-01	Map2k6	0.73	5.75E-01	Tnfsf10	0.73	4.91E-01
Cacna1e	0.61	3.22E-01	Il11ra1	0.61	1.15E-01	Tshr	0.66	5.10E-01	Fgf6	0.72	8.04E-01	Fzd2	0.73	3.48E-01
Mmp9	0.60	3.61E-01	Acvr2a	0.61	1.74E-02	Cacna1e	0.66	4.86E-02	Cxcl2	0.71	8.17E-01	Il23a	0.73	6.94E-01
Rasgrp1	0.60	7.60E-01	Il22ra2	0.60	1.75E-01	Hspb1	0.65	2.37E-01	Gngt1	0.71	8.04E-01	Gadd45g	0.72	6.94E-01
Pla2g3	0.60	8.62E-01	Nr4a1	0.60	2.13E-01	Acvr2a	0.64	5.47E-03	Il1b	0.71	8.02E-01	Gzmb	0.72	6.94E-01
Calml3	0.60	9.66E-01	Comp	0.60	3.13E-01	Ppargc1a	0.64	2.24E-01	Pax5	0.71	8.02E-01	Il3	0.72	8.68E-01
Itga8	0.60	2.03E-01	Wnt7b	0.58	5.35E-01	Fgf13	0.63	3.82E-01	Fgf12	0.69	9.32E-01	Epor	0.72	3.35E-01
Wnt2	0.60	2.40E-01	Ptpn5	0.57	7.72E-02	Pla2g3	0.63	4.87E-01	Cd19	0.69	5.75E-01	Itgb6	0.72	4.91E-01
Bmp7	0.59	2.41E-01	Pparg	0.56	2.69E-01	Ddb2	0.62	4.94E-03	Ntrk2	0.69	8.17E-01	Col3a1	0.71	6.16E-01
Wnt10a	0.59	6.32E-01	Rps6ka6	0.55	7.23E-02	Zbtb16	0.61	3.40E-01	Suv39h2	0.69	8.02E-01	Il1b	0.70	7.48E-01
Flt3	0.58	3.61E-01	Cntfr	0.55	5.98E-02	Vegfb	0.61	2.89E-02	Ntf3	0.68	8.02E-01	Rxrg	0.70	8.68E-01
Gng4	0.57	4.97E-01	Jun	0.54	6.94E-03	Sfrp4	0.61	3.59E-01	Cntfr	0.68	8.02E-01	Sgk2	0.69	9.41E-01
Hspb1	0.57	6.01E-01	Tshr	0.53	4.46E-01	Efna3	0.61	4.48E-01	Ibsp	0.68	8.02E-01	Inhbb	0.69	8.42E-01
Pla2g4e	0.53	8.78E-01	Acvr1c	0.53	2.92E-01	Prkar2b	0.60	2.93E-01	Tnc	0.67	8.02E-01	Mycn	0.69	3.57E-01
Pck1	0.53	9.30E-01	Calml3	0.53	8.12E-01	Pla2g10	0.60	4.11E-01	Nr4a3	0.67	8.02E-01	Il6	0.68	7.48E-01
Wnt7b	0.52	7.66E-01	Ddb2	0.53	5.82E-03	Ccr7	0.58	1.64E-03	Tshr	0.66	9.04E-01	Ccr7	0.68	4.30E-01
Ccr7	0.50	2.66E-02	Figf	0.52	1.96E-03	Lep	0.57	1.90E-01	Comp	0.66	8.04E-01	Hhex	0.66	3.86E-01
Ifng	0.50	3.61E-01	Zbtb16	0.52	3.57E-01	Calml3	0.56	7.10E-01	Klf4	0.64	8.02E-01	Nog	0.65	9.04E-01
Il1r2	0.49	7.60E-01	Pax5	0.51	6.31E-03	Wnt7b	0.55	3.39E-01	Uty	0.63	8.02E-01	Ifna1	0.64	7.48E-01
Rps6ka6	0.49	1.35E-01	Vegfb	0.51	2.24E-02	Hspa1a	0.54	2.58E-01	Prkar2b	0.62	8.08E-01	Zic2	0.64	7.38E-01
Figf	0.48	6.97E-03	Sfrp4	0.48	2.88E-01	Ntrk2	0.54	1.42E-01	Osm	0.61	8.02E-01	Flt3	0.64	6.20E-01
Fgf13	0.47	6.72E-01	Pdgfra	0.47	2.20E-03	Fgfr4	0.53	5.39E-03	Pparg	0.60	8.02E-01	Cd19	0.62	2.34E-01
Efna3	0.47	7.36E-01	Il12a	0.46	2.54E-04	Rps6ka6	0.52	6.54E-03	Pla2g5	0.60	8.02E-01	Prl	0.62	5.18E-01
Fgfr4	0.46	8.93E-02	Ppargc1a	0.46	9.06E-02	Cacng6	0.50	3.28E-01	Fgf21	0.56	8.02E-01	Pax5	0.61	3.57E-01
Ptpn5	0.44	6.04E-02	Ntrk2	0.42	1.14E-01	Figf	0.50	3.94E-05	Ppargc1a	0.51	8.02E-01	Pla2g4e	0.61	9.43E-01
Il12a	0.42	1.09E-03	Klf4	0.42	1.96E-03	Ptpn5	0.50	3.67E-03	Sost	0.50	8.17E-01	Mmp3	0.58	5.82E-01
Pdgfra	0.42	7.79E-03	Cacng1	0.41	6.10E-01	Il12a	0.44	2.62E-06	Fos	0.45	8.02E-01	Wnt7a	0.57	2.13E-02
Pla2g10	0.39	6.39E-01	Cd19	0.40	6.90E-05	Pdgfra	0.44	5.15E-05	Il20ra	0.44	8.02E-01	Lif	0.57	2.13E-02
Igfbp3	0.38	4.96E-02	Prkar2b	0.39	1.12E-01	Pax5	0.41	3.94E-05	Inhba	0.41	8.17E-01	Tnc	0.54	4.28E-01
Pax5	0.33	1.66E-03	Igfbp3	0.37	4.67E-03	Igfbp3	0.38	3.83E-04	Nr4a1	0.38	4.14E-01	Ppp2r2c	0.52	3.19E-01
Cacng1	0.32	8.22E-01	Lep	0.37	8.02E-02	Cacng1	0.36	4.08E-01	Hspa1a	0.38	8.02E-01	Cxcl5	0.49	5.22E-02
Cd19	0.30	7.97E-05	Hspa1a	0.28	7.72E-02	Cd19	0.35	2.67E-07	Sgk2	0.35	8.02E-01	Ifnb1	0.45	7.92E-01
Cacng6	0.28	4.31E-01	Pck1	0.14	2.42E-01	Pck1	0.27	3.30E-01	Pck1	0.17	8.02E-01	Inhba	0.34	7.94E-01

**Table S3 |** Taqman gene expression assays (Life Technologies), related to STAR methods reagents				
**Probe ID**	**Gene**	**Species**	**Full name**	**Reporter**
Mm00446190_m1	*Il6*	Mouse	Interleukin 6	FAM
Mm04207460_m1	*Cxcl-1*	Mouse	Chemokine (C-X-C motif) ligand 1, KC	FAM
Mm00440295_m1	*Mmp3*	Mouse	Matrix metalloproteinase 3	FAM
Mm00441818_m1	*Timp1*	Mouse	TIMP metallopeptidase inhibitor 1	FAM
Mm00494449_m1	*Cdkn2a*	Mouse	CDK inhibitor 2a, p16	FAM
Mm01303209_m1	*Cdkn1a*	Mouse	CDK inhibitor 1a, p21	FAM
Mm00438168_m1	*Cdkn1b*	Mouse	CDK inhibitor 1b, p27	FAM
Mm00439620_m1	*Il1a*	Mouse	Interleukin 1a	FAM
Mm00439560_m1	*Igf1*	Mouse	Insulin-like growth factor 1	FAM
Mm00437762_m1	*B2m*	Mouse	Beta-2-Microglobulin	FAM
4352339e-1207040	*Gapdh*	Mouse	Glyceraldehyde 3 phosphate dehydrogenase	VIC
Mm01309913_m1	*Tmem199*	Mouse	Transmembrane protein 199	FAM
Hs00985639_m1	*IL6*	Human	Interleukin 6	FAM
Hs00174103_m1	*IL8*	Human	Interleukin 8	FAM
Hs00968305_m1	*MMP3*	Human	Matrix metalloproteinase 3	FAM
Hs00171558_m1	*TIMP1*	Human	TIMP metallopeptidase inhibitor 1	FAM
Hs00355782_m1	*CDKN1A*	Human	CDK inhibitor 1a, p21	FAM
Hs00923894_m1	*CDKN2A*	Human	CDK inhibitor 2a, p16	FAM
Hs99999907_m1	*B2M*	Human	Beta-2-Microglobulin	FAM
4310884E	*GAPDH*	Human	Glyceraldehyde 3 phosphate dehydrogenase	VIC

**Table S4 |** Antibodies, related to STAR methods reagents				
**Antibody Target**	**Source**	**ID**	**Species**	**Dilution**
p21	Dako	M7202	Mouse	1/50
p21	Abcam	ab188224	Rabbit	1/4000
γH2Ax (S139)	Cell Signaling	9718S	Rabbit	1/100
α Smooth muscle actin	Sigma	A2547	Mouse	1/5000
EpCAM	Abcam	ab221552	Rabbit	1/200
Endomucin (V7C7)	Santa Cruz	sc65495	Rat	1/1000
CD4	Ebioscience	14-9766	Rat	1/250
CD8	Ebioscience	14-0808	Rat	1/250
Ki67	Abcam	ab16667	Rabbit	1/300
MCL-1	Cell Signaling	94296	Rabbit	1/80
Firefly luciferase	Abcam	ab181640	Goat	1/100
Lamin A/C	Abcam	ab108595	Rabbit	1/1000
Mouse IgG-488	Invitrogen	A11001	Goat	1/1000
Mouse IgG-555	Invitrogen	A21127	Goat	1/1000
Rat IgG-488	Invitrogen	A11006	Goat	1/1000
Rabbit IgG-488	Invitrogen	A11008	Goat	1/1000
IgG, immunoglobulin G; γH2Ax, phosphorylated histone H2AX				
